# Vices and vegetables: a systematic review and series of meta-analyses examining the relationships among compensatory health beliefs with health-related intentions and behaviors

**DOI:** 10.1093/abm/kaag013

**Published:** 2026-04-19

**Authors:** Gavin Q Fox, Minh Quang Luong, Allison Kimmel, Lucy E Napper

**Affiliations:** Department of Psychology, Lehigh University, Bethlehem, PA 18015, United States; Health, Medicine, and Society Program, Lehigh University, Bethlehem, PA 18015, United States; Department of Psychology, Lehigh University, Bethlehem, PA 18015, United States; Department of Psychology, Lehigh University, Bethlehem, PA 18015, United States; Department of Psychology, Lehigh University, Bethlehem, PA 18015, United States; Health, Medicine, and Society Program, Lehigh University, Bethlehem, PA 18015, United States

**Keywords:** compensatory health beliefs (CHBs), health behavior, intentions, meta-analysis, justification, compensatory behavior

## Abstract

**Background:**

Compensatory health beliefs (CHBs), the belief that a healthy behavior can offset the effects of a health-compromising one, have been theorized to both justify unhealthy behaviors and motivate health-promoting ones. However, findings in the literature are inconsistent.

**Methods:**

These meta-analyses synthesized 252 effect sizes from 59 independent samples using robust variance estimation to examine the relationships between CHBs and four outcomes: intentions and engagement in compensatory health behaviors and intentions and engagement in health-compromising behaviors.

**Results:**

Approximately 13,760 individuals were represented across these analyses (ages 12-79; women ∼66.1%; college-based = 69.8%). CHBs were significantly associated with greater intentions to engage in compensatory health behaviors (*r* = 0.21) and with greater engagement in health-compromising behaviors (*r* = 0.18), but were not reliably associated with compensatory health behaviors or intentions to engage in health-compromising behaviors. Heterogeneity was substantial across all models, but only one significant moderator emerged: CHBs were more strongly associated with compensatory intentions in studies examining diet compared to physical activity. Funnel plot symmetry and stability across values of ρ supported the robustness of findings.

**Conclusions:**

These results highlight the potential for CHBs to undermine health behavior change efforts, particularly when intentions to engage in healthy behaviors are not carried out. Future work should explore state-level CHBs and real-time decision-making to clarify how and when these beliefs influence behavior.

## Teaser

In our everyday lives, we frequently justify our unhealthy decisions by convincing ourselves that we can cancel out the negative effects of an unhealthy behavior by engaging in a healthy behavior. This paper demonstrates that while these beliefs are associated with good intentions, people rarely follow through with the healthy behavior despite using these beliefs to justify their unhealthy ones.

Health-compromising behaviors (ie, engaging in unhealthy behaviors and avoiding healthy ones) are one of the strongest predictors of developing noncommunicable diseases.^1^^,^^2^ Therefore, understanding the psychological mechanisms that influence these behaviors is essential. Compensatory health beliefs (CHBs),^3^^,^^4^ or the belief that engaging in a healthy behavior can make up for a health-compromising behavior, is one such mechanism. CHBs, such as, “It is OK if I eat a donut, because I will make-up for it by exercising later”, can be used to justify health-compromising behaviors, leading to the persistence of unhealthy behaviors despite awareness of their risks.^4^ Additionally, under certain circumstances, CHBs may motivate people to engage in compensatory health behaviors (eg, exercising, maintaining a healthy diet) in order to counteract the negative effects of unhealthy choices.^5^ While some healthy behaviors can reduce the negative effects of unhealthy ones, compensation is not always able to fully or safely counteract the negative effects of all health-compromising behaviors (eg, smoking,^6^ excessive alcohol use^7^) highlighting the importance of research on the relationships between CHBs and behavioral outcomes. Although many studies have examined how CHBs relate to intentions and behaviors across healthy and unhealthy behavioral domains, mixed findings highlight the need for meta-analyses to clarify when and how these associations emerge.

## Compensatory Health Belief Model (CHBM)

The Compensatory Health Belief Model (CHBM)^4^ outlines the importance of CHBs for understanding behavior. According to the CHBM, CHBs are activated when a person encounters a tempting situation that conflicts with a higher order goal, for example, the goal of being healthy. CHBs can help reduce dissonance experienced in these situations by providing a means of justifying behaviors that do not align with a long-term health goal.^4^^,^^8^^,^^9^ Therefore, the activation of CHBs should immediately lead to the formation of an intention to engage in both a health-compromising behavior and a compensatory health behavior that can cancel out the negative effects of the unhealthy behavior. In addition to being central to the CHBM, CHBs have received renewed attention in research on multiple health behavior change.^10^ The Compensatory Carry-over Action Model (CCAM)^5^ builds on the CHBM by emphasizing how CHBs (or compensatory cognitions), can facilitate or hinder progress across interrelated health behaviors. This underscores the importance of better understanding CHBs not just for single health behaviors, but also within the context of how individuals manage multiple health goals simultaneously and how interventions might support multiple health behavior change by addressing these beliefs.

Consistent with the CHBM, some studies have found that CHBs are associated with stronger intentions to engage in health-compromising behaviors, such as alcohol consumption^11^ and an unhealthy diet;^12^ as well as stronger intentions to engage in healthy behaviors one believes can make-up for health-compromising ones, such as regular physical activity and a healthy diet.^13–16^ In contrast, other studies have found CHBs are associated with weaker intentions to engage in health behaviors.^17–19^ Given these mixed findings, the current meta-analyses are designed to clarify the relationships between CHBs and intentions to engage in health-compromising and compensatory health behaviors across contexts.

While intentions represent an important step toward behavior change, it is equally important to consider whether CHBs play a role in actual behavior. Given that CHBs increase intentions to act, it is perhaps not surprising that CHBs are theorized to be positively associated with both health-compromising behaviors and compensatory health behaviors.^4^^,^^5^ If CHBs are activated post hoc to justify an unhealthy action that has already occurred, these beliefs should be positively associated with health-compromising behaviors.^4^^,^^5^ In contrast, there are a number of reasons why CHBs may not be associated with compensatory health behaviors. First, it is possible that simply forming an intention to compensate for an unhealthy behavior relieves feelings of dissonance even if the compensatory health behavior is never performed. Similarly, a compensatory health behavior may no longer be necessary if one reduces their dissonance in a different way, such as engaging in another healthy behavior.^4^ Lastly, even if the dissonance still exists, and one has strong intentions to engage in a compensatory health behavior to resolve this dissonance, people often fail to translate their intentions into actions.^20^

Studies examining the relationships among CHBs with healthy and unhealthy behaviors have produced mixed results. Specifically, some studies have found that CHBs are positively correlated with health-compromising behaviors.^3^^,^^21^^,^^22^ In terms of compensatory health behaviors, some work suggests that CHBs are negatively correlated,^3^^,^^18^^,^^23^^,^^24^ or positively correlated, with healthy behaviors.^13^^,^^25–27^ Lastly, other work suggests that CHBs may not be associated with (un)healthy behaviors.^13^^,^^28^^,^^29^ Taken together, the inconsistent associations among CHBs with compensatory health, and health-compromising, behaviors underscores the importance of the current meta-analyses in clarifying these relationships and identifying potential moderators that may account for variability in effects across studies.

## Potential moderators of CHBs

### Self-efficacy

The CHBM posits that self-efficacy may moderate the relationship between CHBs and compensatory health behaviors as well as health-compromising ones.^4^ For example, if someone is confident in their ability to perform a healthy behavior, they will carry out this behavior even if they believe it could be compensated for. Similarly, if someone has high self-efficacy to perform a compensatory health behavior (eg, physical activity), the compensatory belief that physical activity can make up for a poor diet should be associated with greater intentions to engage in physical activity. In the case of health-compromising behaviors, one study found that when self-efficacy to eat fruits and vegetables was low, there was a negative relationship between participants’ beliefs that they could compensate for their poor diet with other healthy behaviors and intentions to eat fruits and vegetables.^30^ There was no such relationship when self-efficacy was moderate-to-high. While few studies have tested self-efficacy as a moderator of the CHB-intention relationships, to our knowledge, none have examined self-efficacy as a moderator of the CHB-behavior relationships. Considering that self-efficacy may play an important role in the relationships among CHBs with intentions and behavior, in the current study we explored whether the strength of these relationships across studies was moderated by self-efficacy.

### Risk perception

Another potential moderator of the relationships among CHBs with intentions to engage, and actual engagement, in health behaviors is the perceived risk to one’s health as a result of health-compromising behaviors.^4^^,^^31^ CHBs are only likely to motivate a healthy behavior if the individual believes the unhealthy behavior poses a serious threat to their health.^4^ In line with this prediction, previous research has found that there is a positive relationship between CHBs and intentions to diet when perceived risk was medium-to-high, but not low.^15^^,^^19^ Given this research, we explored whether perceived risk moderated the CHB-intention and CHB-behavior relationships in the current study.

### Time (cross-sectional vs. longitudinal)

CHBs should have the strongest association with intentions in the moment that they are activated in order to combat dissonance, with this association becoming weaker over time.^4^ This is true both for compensatory health behaviors and health-compromising behaviors. For example, in the case of health-compromising behaviors, if the tempting behavior is not performed immediately after activating the CHB, the temptation may pass. Similarly, the relationships among CHBs and compensatory intentions/behavior should get weaker over time as dissonance fades and people lose motivation or forget to perform the compensatory health behavior.^4^^,^^10^^,^^22^^,^^32^ Given that research on CHBs has used a variety of time periods for studying these associations, examining time as a moderator of these relationships may help elucidate the mixed findings in the current literature.

### Measurement of CHBs (behavior-specific vs. general)

Another potential factor that could explain different patterns of results across studies is variations in how CHBs are measured. While some studies use the original 17-item CHB scale,^3^ which assesses the belief that a range of behaviors can compensate for one another, others have developed CHB measures that are specific to a single compensatory and/or health-compromising behavior. For example, a measure of smoking-specific CHBs^31^ assesses how a range of healthy behaviors, like physical activity, a healthy diet, reduced alcohol use, and limiting future smoking, can compensate for the single health-compromising behavior of smoking. Constructs that are measured using behavior-specific items, rather than general items, should be better predictors of behavioral outcomes.^33–35^ While some studies have included both specific and general measures of CHBs,^29^^,^^36–39^ to our knowledge, no one has directly tested differences in the strengths of the associations among the CHB-intention and CHB-behavior relationships as a function of measure specificity. The current study adds to the literature by examining whether there is a difference in effect sizes among CHBs with behavioral outcomes as a function of whether CHBs are specific to a single behavioral outcome being studied or refer to a variety of different behaviors.

### Behavior type (physical activity vs. diet)

In addition to the specificity of the measurement approach used, past research also varies based on the type of health behavior studied (eg, physical activity, diet, medication use). It is possible that CHBs are a better predictor of some health behaviors than others.^19^ For example, Forestier and colleagues^19^ found that the belief that physical activity could compensate for diet was negatively associated with diet intentions, however the belief that diet could compensate for physical activity was not associated with physical activity intentions. Differences in relationships across behaviors may reflect people being less willing to engage in healthy behaviors that they perceive as high effort when they believe there is a less effortful behavior(s) that can achieve the same health goal.^19^^,^^40^ Much of the research on CHBs examines physical activity and diet, therefore, we explored whether the relationships among CHBs with intentions and behaviors differed for physical activity vs diet.

## Current study

The present study builds on prior work by systematically examining the relationships among compensatory health beliefs (CHBs) with intentions to engage, and actual engagement, in health behaviors. Specifically, we differentiate between four types of outcomes theorized in the CHBM: intentions to perform a health-compromising behavior that can be compensated for (eg, intending to eat a donut or delay exercise); health-compromising behaviors, which are the actual enactment of those unhealthy behaviors (eg, eating a donut) or refraining from a healthy behavior (eg, skipping exercise); compensatory intentions to perform a healthy behavior meant to offset the effects of an unhealthy behavior (eg, intending to exercise later to make up for overeating); and compensatory health behaviors that one believes can make-up for the lack of another healthy behavior or that can cancel out the negative effects of an unhealthy behavior (eg, actually engaging in exercise after eating a donut). These distinctions are important, as prior studies often conflate, or only examine one, of these categories, potentially obscuring important differences in how CHBs relate to health outcomes.^10^^,^^19^ Based on the CHBM and prior empirical findings, we hypothesized that CHBs would be positively associated with both intentions to engage in health-compromising behaviors and compensatory health behaviors that can cancel them out. However, we expected these intentions would not translate equally into action. Specifically, we hypothesized that CHBs would be positively associated with health-compromising behaviors, but not with compensatory health behaviors. This prediction reflects the ideas that CHBs are often activated post hoc, either to justify or reduce dissonance about an unhealthy action, and that individuals frequently fail to follow through on prospective compensatory intentions.^4^^,^^22^^,^^32^ Thus, although CHBs may motivate intentions to behave healthily in the future, they may not reliably predict actual, healthy behavior. Supplementary Figure 1 depicts the hypothesized relationships among CHBs with intentions and behaviors.

Next, we examined moderators of the relationships among CHBs, intentions, and behaviors. Regarding the role of self-efficacy, we hypothesized that the CHB-intention and CHB-behavior relationships would be stronger when self-efficacy to engage in behaviors was high compared to low.^4^^,^^5^ Additionally, we expected CHBs to be more strongly positively associated with behavioral outcomes at high, compared to low, levels of perceived health risk.^15^^,^^19^^,^^31^ With respect to time, we predicted that the relationships among CHBs with intentions and behaviors would be weaker for longitudinal studies compared to cross-sectional studies.^4^^,^^10^^,^^22^^,^^32^ In regard to the specificity with which CHBs were measured, we anticipated that when CHBs were assessed at a behavior-specific, as opposed to a general, level the relationships among CHBs with intentions and behaviors would be stronger. Finally, we explored whether the relationships among CHBs with intentions and behavior were moderated by the type of behavior (ie, physical activity vs diet). Given the exploratory nature of this analysis, we did not make specific predictions about the direction of these effects.

## Methods

This paper follows the best practices of meta-analyses outlined in PRISMA^41^ and MARS.^42^

### Eligibility criteria

Studies were included in the meta-analysis if they met all of the following criteria:

- Topic: The study examined CHBs or compensatory cognitions and their relationship to health-related behavioral intentions and/or actual health behaviors.
- Measurement: CHBs had to be measured using a version of the Compensatory Health Beliefs Scale or an equivalent measure assessing beliefs that health behavior(s) could cancel out the effects of a health-compromising behavior(s). Data based on behavior-specific CHBs were only included if a relevant corresponding compensatory or health-compromising behavior was also assessed.
- Health Focus: The study must have focused on individual-level health behavior (eg, physical activity, diet, alcohol use) rather than environmental, political, or parenting contexts (eg, parental beliefs about a child’s health behavior).
- Empirical Data: The study must have been empirical (ie, reporting original data) and reported sufficient statistical information (eg, correlation coefficients, sample sizes) to compute effect sizes or provided this information upon request.
- Language: Studies were required to be written in English (though within these studies measures were administered in multiple languages, including Dutch, German, Chinese, Italian, Greek, and Spanish).
- Design: Both cross-sectional and longitudinal designs were eligible. There was no requirement for temporal ordering (eg, in one study CHBs were measured after intentions). If CHBs were manipulated, the control group was used, or if a control group was not available and CHBs did not significantly decrease as a result of the manipulation, then the study was retained.
- Publication Status and Sample Size: There was no minimum sample size requirement and studies were included regardless of publication status. Papers published in multiple places and those reporting the same effects were excluded (eg, papers also published in dissertations or student papers written on the same topic and sample).

**Figure 1:**
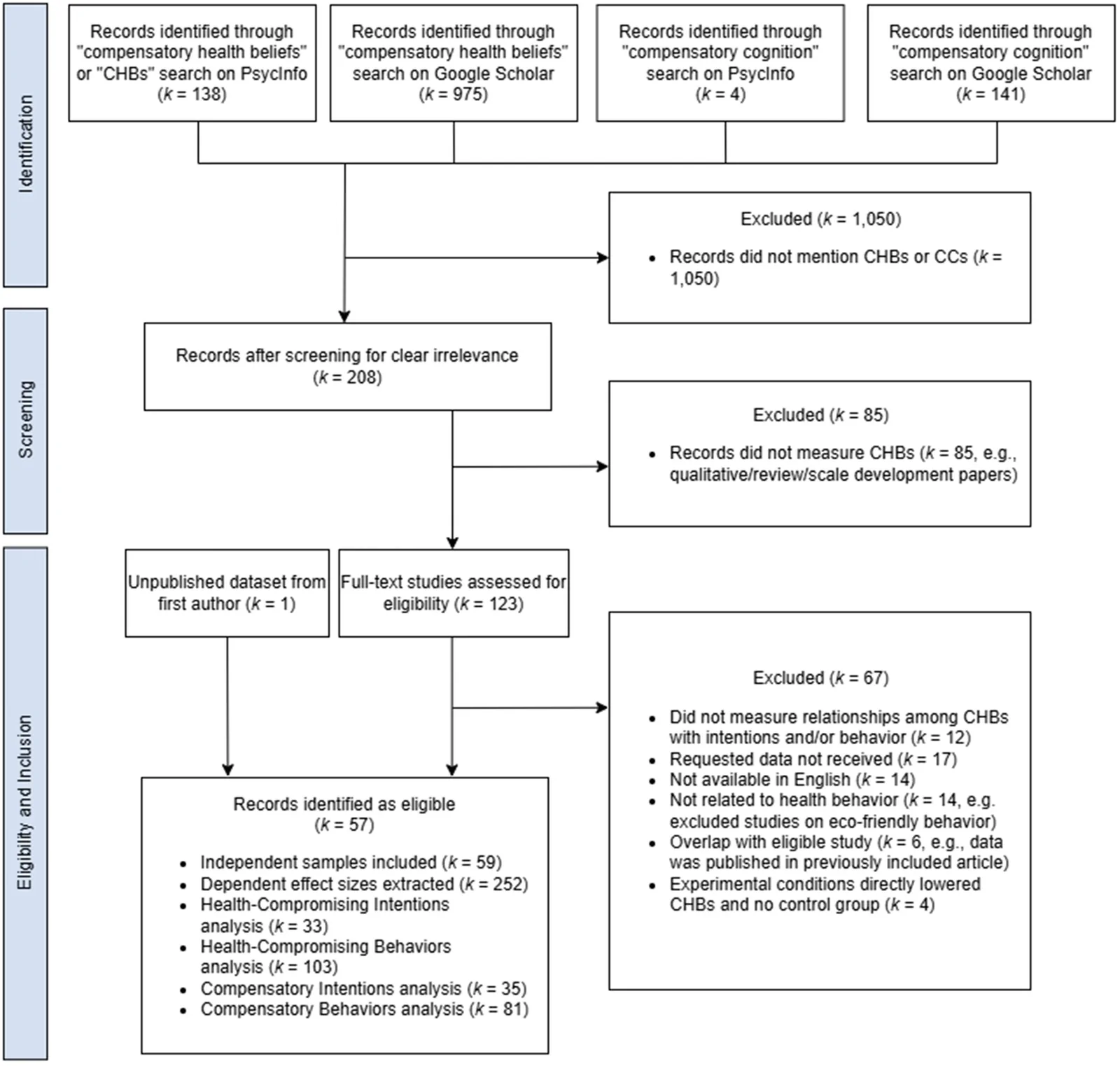
PRISMA flowchart for study inclusion and exclusion.^41^

Full details of the inclusion and exclusion process are provided in the PRISMA flowchart for inclusion (see Figure 1).

### Search strategy

A systematic search was conducted using two electronic databases and supplementary methods:

- **PsycINFO**: 138 records using the keywords “compensatory health beliefs” OR “CHBs” and 4 records with “compensatory cognition.”
- **Google Scholar**: 975 records for “compensatory health beliefs” OR “CHBs,” and 141 records for “compensatory cognition.”

The search began on July 15, 2024, and concluded on February 23, 2025. Publications from all years were accepted with the earliest eligible publication being from 2004, and the most recent from 2025. In addition to the database search, we contacted corresponding authors of potentially eligible studies to request data not described in a paper. We also included an unpublished study from the first author and contacted any author who had published three or more qualifying studies to inquire about unpublished data or additional studies.

### Study selection

Titles and abstracts were screened independently by the first three authors. Full texts of potentially eligible studies were then reviewed to determine final inclusion.

### Data extraction

For each study, the following information was extracted when possible:

1. Citation and publication status
2. Country where the study was conducted
3. Sample characteristics (eg, age, sex distribution)
4. Type of CHBs assessed and measure used (ie, behavior-specific or general)
5. Reliability of the CHBs scale used (ie, Cronbach’s alpha)
6. Type of health behavior(s) (eg, physical activity, diet, alcohol use)
7. Outcome type (health-compromising and/or compensatory; intentions and/or behaviors)
8. Effect size information (correlation coefficients, sample sizes)
9. Type of design (ie, cross-sectional or longitudinal)
10. Mean, SD, and range of values of moderators

Where studies reported multiple relevant effects, all eligible effects were included and modeled using Robust Variance Estimation (RVE) to account for dependency. Data were extracted by the first three authors and reviewed by the fourth author to ensure accuracy and resolve ambiguities. Any issues were resolved by consensus between the first and fourth authors.

### Behavior coding and effect direction

When examining CHBs regarding how two or more behaviors were related to each other, a behavior was coded as a health-compromising behavior when CHB items indicated that the behavior was being compensated for, and as a compensatory health behavior when the wording of CHB items indicated that the behavior was intended to cancel out a health-compromising behavior.

To ease interpretability, effect sizes for health-compromising behaviors were coded to ensure that a positive mean effect indicated that CHBs are associated with more unhealthy intentions/behaviors or less healthy intentions/behaviors. Conversely, in the analyses of compensatory health behaviors, a positive mean effect indicates that CHBs are associated with more healthy intentions/behaviors or less unhealthy intentions/behaviors.

In situations where CHBs were defined as the same behavior making up for itself at a later time (eg, making up for not exercising today by exercising tomorrow; eating less unhealthy food tomorrow so you can eat more unhealthy food today), the behavior was considered as both a health-compromising and a compensatory health behavior and the effect size of such relationships may appear in more than one analysis.

Throughout the studies we examined, self-efficacy was defined in several ways. If self-efficacy was a general construct or specific to a target behavior it was included as a moderator (eg, we did not include physical activity self-efficacy as a moderator of the relationship between CHBs and diet behavior). In addition to being defined as confidence in one’s ability to carry out a (un)healthy behavior, throughout our review we also found that self-efficacy could be defined as one’s ability to resist engaging in a tempting behavior (eg, resisting drinking). Conversely, other studies examined self-efficacy in relation to a healthy behavior that one believes can be compensated for. Given that in the latter two cases, we would expect the relationships among CHBs with intentions and behaviors to get weaker at high levels of self-efficacy, we ran separate analyses distinguishing between the different types of self-efficacy, in addition to including all types of self-efficacy in the main analysis. The pattern of results for these separate analyses did not differ and, therefore, only the main analysis is reported in text.

### Analysis plan

#### Data preparation

We extracted 252 effect sizes (correlations) from 59 independent samples and transformed these scores to Fisher’s *Z* to ensure normality and comparability across studies.^43^ Violin plots were used to assess potential outliers in effect sizes, defined as values exceeding 1.5 times the interquartile range beyond the quartiles. No such outliers were detected prior to synthesizing the data.

#### Robust variance estimation (RVE)

Standard meta-analysis techniques ignore the complexities of dependent data structures, which can result in inflated estimates of the average effect size and artificially narrow confidence intervals.^44^^,^^45^ Therefore, we employed RVE with ρ = 0.8 using the robumeta package in R version 4.2.2 to calculate our four primary mean effects, meta-regression models, and forest plots.^45^^,^^46^ As recommended, we applied the small-sample correction to adjust the residuals and degrees of freedom in all of our analyses^47^ and report these along with their 95% confidence intervals (CIs). We used a random-effects model given that studies in the literature vary widely in terms of population and methodology, and thus the relationships between CHBs and outcomes are expected to differ across studies.^48^ This approach allows us to generalize findings to comparable future populations and settings.^49^^,^^50^

#### Primary effect estimation

Four meta-analyses were conducted to estimate the mean effect sizes for the relationships between CHBs and four outcomes: intentions and engagement in health-compromising behaviors and intentions and engagement in compensatory health behaviors. Forest plots were generated for the four primary analyses to visualize the distribution and consistency of effects. We included *I*^2^ as a measure of the proportion of variance in observed effects that is due to true differences in effect sizes rather than sampling error, where *I*^2^ > 50% indicates notable heterogeneity and *I*^2^ < 30% indicates mild heterogeneity.^51^^,^^52^ We employed τ^2^ as an indicator of unexplained variance in these true effects relative to the metric of the effect size used.^48^ Prediction intervals were calculated for the four primary analyses to assist in the interpretation of between-study variance given that there were more than five studies in each analysis and there was no funnel plot asymmetry.^53–55^ These intervals represent the range of effects within which the true effect of a future similar study is likely to fall.^56^

#### Bias and sensitivity assessment

Studies that find significant results or support for prominent theories are more likely to be published,^57^ introducing potential bias. To minimize this, we included eligible studies regardless of publication status. Additionally, funnel plots were used to assess the presence of publication bias, although it should be noted that funnel plot asymmetry could also be due to other factors (eg, small-study effects, heterogeneity).^58^ Asymmetric plots suggest the absence of studies with smaller effect sizes, indicating bias, while symmetric plots indicate variability due to sampling error.^58–61^ Risk of other biases introduced in individual studies was not independently assessed using a formal tool. However, potential measurement quality issues were used as a measure of bias in individual studies and were addressed through moderator analysis of the internal consistency (Cronbach’s alpha) of the CHB scale used in each study (see Table 1). Sensitivity analyses were also conducted to examine if τ^2^ and the average effect size in each primary analysis differed as a function of rho (ρ), or how much variance was shared among effects within the same study (eg, 0 = completely independent; 1 = completely dependent). Given that ρ is an estimate of the relationships among effect sizes within a study and influences standard errors, it was important to make sure that our results were significant regardless of how closely effects within a study were actually related.

**Table 1:** Summary statistics.

Analysis	# of studies	# of outcomes	*z/b*	*t*	*df*	*P*	CI lower	CI upper	τ2
**Health-compromising intentions**	**17**	**33**	**0.079**	**1.33**	**15.8**	**.203**	**−0.047**	**0.205**	**0.080**
Time	17	33	0.224	1.49	4.39	.203	**−**0.178	0.625	0.046
Self-efficacy	14	30	0.008	1.53	4.62	.192	**−**0.006	0.022	0.031
Risk perception	6	10	0.009	3.16	2.48	.066	**−**0.001	0.020	0.072
CHB-type	17	33	0.129	2.09	15	.054	**−**0.003	0.261	0.080
Behavior type	11	27	**−**0.037	**−**0.43	4.16	.690	**−**0.274	0.200	0.021
Alpha	11	23	**−**0.098	**−**0.13	3.17	.908	**−**2.510	2.310	0.048
**Health-compromising behaviors**	**45**	**103**	0.184***	**4.89**	**43.4**	**<.001**	**0.108**	**0.260**	**0.056**
Time	45	103	**−**0.070	**−**1.07	13.2	.304	**−**0.212	0.071	0.057
Self-efficacy	19	51	0.001	0.35	4.81	.744	**−**0.009	0.011	0.076
Risk perception	6	9	0.011	2.22	2.63	.125	**−**0.006	0.028	0.012
CHB-type	45	103	**−**0.123	**−**2.10	11.68	.058	**−**0.251	0.005	0.057
Behavior type	28	55	0.026	0.34	16.43	.735	**−**0.135	0.188	0.049
Alpha	36	90	0.351	0.92	10.56	.379	**−**0.494	1.195	0.052
**Compensatory intentions**	**16**	**35**	0.210**	**3.11**	**14.8**	**.007**	**0.066**	**0.354**	**0.071**
Time	16	35	**−**0.157	**−**1.57	6.11	.167	**−**0.400	0.086	0.067
Self-efficacy	9	25	0.000	**−**0.03	3.06	.975	**−**0.024	0.025	0.052
Risk perception	4	5	0.018**	15.90	1.94	.005	0.013	0.023	0.000
CHB-type	16	35	**−**0.025	**−**0.16	12.68	.875	**−**0.363	0.313	0.079
Behavior type	11	27	0.133*	2.48	8.89	.036	0.011	0.255	0.022
Alpha	15	34	0.451	1.12	3.67	.332	**−**0.712	1.615	0.066
**Compensatory behaviors**	**30**	**81**	**0.098**	**2.01**	**28.8**	**.053**	**−0.002**	**0.198**	**0.077**
Time	30	81	**−**0.011	**−**0.08	6.14	.941	**−**0.372	0.349	0.078
Self-efficacy	11	31	**−**0.003	**−**0.52	3.57	.636	**−**0.022	0.015	0.082
Risk perception	4	5	**−**0.002	**−**0.36	1.98	.776	**−**0.029	0.025	0.051
CHB-type	30	81	0.042	0.37	18.08	.717	**−**0.196	0.279	0.080
Behavior type	23	48	**−**0.048	**−**0.43	20.8	.669	**−**0.281	0.184	0.085
Alpha	28	78	0.482	0.93	7.34	.382	**−**0.732	1.696	0.074

CI = confidence interval; *df* = degrees of freedom; CHB = compensatory health beliefs. # = Number. For Time; 0 = Cross-sectional; 1 = Longitudinal. For CHB-Type; 0 = General; 1 = Specific. For Behavior Type; 0 = Physical Activity; 1 = Diet; Alpha = Cronbach’s α. Summary/mean effects are indicated in bold; for these, correlations (Fisher’s z) are reported. All other effects are unstandardized regression coefficients (*bs*). *P* < .05 = *; *P* < .01 = **; *P* < .001 = ***.

#### Meta-regression models

Most studies in these meta-analyses did not include data on multiple moderators. Given that RVE requires complete data for all included moderators and is sensitive to missingness, we conducted meta-regression analyses with one moderator entered at a time and excluded studies missing the relevant data for each analysis. Moderation was considered significant when the *t*-test for the moderator was significant (*P* < .05) and *df* > 4.

#### Moderator variable scaling

We extracted the mean levels of self-efficacy and risk perception as continuous moderators. In line with recommendations from Lipsey and Wilson^43^ to standardize variables across scales while preserving the original distributions and ensuring comparability between moderators, each moderator value was rescaled to fall between 0 and 100, using the following formula:


$$\frac{\left(\mathrm{Observed}\;\mathrm{Score}\;-\;\mathrm{Minimum}\;\mathrm{Possible}\;\mathrm{Score}\right)}{\left(\mathrm{Maximum}\;\mathrm{Possible}\;\mathrm{Score}\;-\;\mathrm{Minimum}\;\mathrm{Possible}\;\mathrm{Score}\right)}\times \;100$$

Where necessary, values were reverse-coded by subtracting the score from 100 so that higher values consistently reflected greater self-efficacy and risk perception.

## Results

### Primary meta-analyses

A wide range of behaviors were captured in the process of this synthesis, ranging from vaccination to safe driving practices (see Supplementary Tables 1-4 for a full list of study characteristics). The most commonly observed behaviors were captured by diet (34.9%) and physical activity (27.4%). The majority of effect sizes were extracted from student samples (71.4%; general population = 23%; clinical = 5.6%) and captured the relationships among CHBs and behaviors (73%) as opposed to intentions (27%). In addition, most studies measured CHBs and behavioral outcomes at the same time (78.6%), as opposed to longitudinally (21.4%), and used behavior-specific (80.6%), rather than general (19.4%), measures of CHBs. Notably, 24 of the included studies were theses or dissertations and thus did not undergo peer review, with 15 of these papers coming from the same university. This study was not pre-registered, however all data files, syntax, results, and supplementary materials (including funnel and forest plots, a conceptual figure, references for included studies, and any information described in the data extraction section) are available on this project’s OSF page: https://osf.io/5sn93.

#### Intentions to engage in health-compromising behaviors

In order to examine the relationship between CHBs and intentions to engage in health-compromising behaviors, 17 studies and 33 outcomes were analyzed. The overall mean effect size was *z *= 0.079, 95% CIs [−0.047, 0.205], *P* = .203, corresponding to an *r* of 0.079, suggesting that CHBs were not reliably associated with intentions to engage in health-compromising behaviors. Between-study heterogeneity was substantial, with *I*^2^ = 95.26% and τ^2^ = 0.080. For CHBs and intentions to engage in a health-compromising behavior, the prediction interval ranged from −0.488 to 0.598.

#### Engagement in health-compromising behaviors

In contrast, 45 studies with 103 outcomes examining CHBs and engagement in health-compromising behaviors revealed a statistically significant positive relationship: *z *= 0.184, 95% CIs [0.108, 0.260], *P* < .001, *r* = 0.182. Individuals with stronger CHBs were more likely to engage in health-compromising behaviors. Heterogeneity was notable, with *I*^2^ = 91.58% and τ^2^ = 0.056. The observed prediction interval for health-compromising behaviors was [−0.291, 0.584].

#### Intentions to engage in compensatory health behaviors

Next, 35 outcomes from 16 studies were included in the analysis of CHBs and intentions to engage in compensatory health behaviors. A significant positive effect was observed, *z *= 0.210, 95% CIs [0.066, 0.354], *P* = .007, *r* = 0.207, indicating that individuals endorsing stronger CHBs were more likely to intend to engage in future health-promoting behaviors. Heterogeneity was also notable, *I*^2^ = 94.05% and τ^2^ = 0.071. The prediction interval for compensatory intentions ranged from −0.359 to 0.662.

#### Engagement in compensatory health behaviors

Finally, 81 outcomes from 30 studies examined CHBs and engagement in compensatory health behaviors. This analysis approached, but did not reach, significance: *z *= 0.098, 95% CIs [−0.002, 0.198], *P* = .053, *r* = 0.097. Heterogeneity was again moderate, *I*^2^ = 94.26%, τ^2^ = 0.077. The prediction interval for compensatory health behaviors spanned −0.444 to 0.587.

Across all four primary analyses, *I^2^* values were consistently high (*I^2^s* > 91%), which indicates that there is substantial heterogeneity in observed effects that is due to true effects rather than sampling error. Additionally, in this study, all τ^2^ values (ranging from ∼0.056 to ∼0.080) were notable, suggesting that moderators can be included to help account for variability in effect sizes within the CHB-intention and CHB-behavior relationships. Lastly, the wide prediction intervals across analyses also indicates substantial variability between studies, with future research on CHBs likely to observe both positive and negative effects regardless of the behavioral outcome.

### Assessment of bias and sensitivity

Visual inspection of funnel plots suggested no evidence of publication bias, with relatively symmetric distributions around the mean effect size for each analysis (see Supplementary Figure 2). To assess whether measurement quality impacted results, internal consistency of the CHB scale (Cronbach’s alpha) was included as a continuous moderator. These analyses indicated that measurement reliability did not explain heterogeneity in effect sizes, *p*s > 0.332. To test the robustness of findings regarding assumptions about the degree of dependence among effect sizes, sensitivity analyses were conducted across different values of the assumed within-study correlation (ρ). These analyses revealed that the average effect sizes and τ^2^ estimates were stable across all values of ρ tested, supporting the robustness of the findings, thus results are reported with ρ at 0.8.

### Moderator analyses

**Figure 2:**
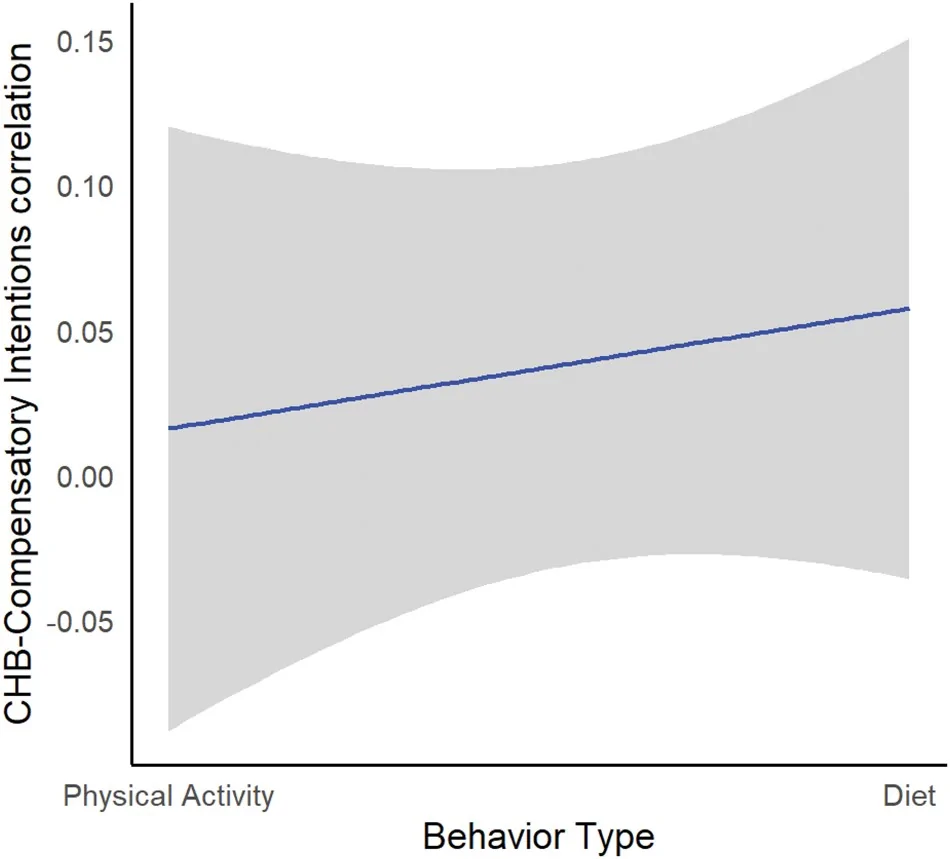
Relationship between CHBs and compensatory intentions moderated by behavior type. The correlation for this graph is in terms of Fisher’s z. The graph is zoomed in to show the effect and thus does not represent the entire range of possible effect sizes.

Only one significant moderation effect emerged (see Table 1 for results). In the analysis of CHBs and intentions to engage in compensatory health behaviors, behavior type significantly moderated the relationship, such that CHBs were more strongly, positively related to compensatory intentions in the context of diet than physical activity, *b *= 0.133, *t*(8.89) = 2.475, *P *= .036, CIs [0.011, 0.255] (see Figure 2).

## Discussion

The present meta-analyses aimed to clarify the relationships among compensatory health beliefs (CHBs) with health-related intentions and health behaviors, while identifying key gaps in the existing literature. Prior studies have primarily focused on diet and physical activity, often examining them as both health-promoting and health-compromising behaviors, with additional research examining risk behaviors such as alcohol use and smoking. Although nearly 60 studies have explored CHBs in relation to intentions and behaviors, few have differentiated between whether the measured outcome reflects a health-compromising behavior (eg, an unhealthy or avoided behavior) or the compensatory health behavior (eg, a healthy action performed to offset the effects of a health-compromising behavior). Instead, most research conceptualizes CHBs broadly as predictors of either healthy or unhealthy behaviors, obscuring the more nuanced distinctions theorized in the CHBM. Our review highlighted that the direction and meaning of these associations depend heavily on how CHBs and outcomes are framed.

### CHBs and health-compromising intentions versus behaviors

In partial support of our hypotheses regarding the relationships among CHBs with health-compromising intentions and behaviors we found that, overall, CHBs were significantly and positively associated with health-compromising behaviors; however, counter to predictions, CHBs were not associated with intentions to engage in health-compromising behaviors. According to the CCAM,^5^ CHBs may emerge either as preparatory justifications or post hoc rationalizations for a health-compromising behavior. In the former case, we would expect CHBs would be associated with greater health-compromising intentions and behaviors. However, when CHBs are formed after the health-compromising behavior has already occurred, we would expect CHBs to be positively associated with health-compromising behaviors, but not intentions. The current analyses demonstrated a pattern of results consistent with the latter case. This finding may reflect the nature of the studies reviewed which included few lab-based, experimental designs that manipulated CHBs.^15^^,^^22^^,^^26^^,^^62–66^ Experimental designs that assess CHBs and intentions after manipulating the presence of a temptation (but prior to being able to act on a temptation) may be better suited for establishing if CHBs are associated with intentions to perform a health-compromising behavior.^22^ Further, few studies identified included ecological momentary assessment designs,^14^^,^^21^^,^^66–68^ where participants are asked to report intentions and behaviors at the moment when CHBs naturally arise. Future work using this approach could also help clarify the relationships among CHBs with intentions.

The positive association of CHBs with health-compromising behaviors highlights that interventions targeting health behaviors may be enhanced by helping people recognize CHBs and their potential to be counterproductive. However, there is limited research on manipulating CHBs and of the previously used methods, efforts to target CHBs have had limited effect.^15^^,^^22^^,^^26^^,^^62–66^ It is possible that approaches such as Cognitive Behavioral Therapy,^69^ including the use of cognitive restructuring, could be used to encourage individuals to monitor their CHBs, question the benefit of these beliefs, and inhibit engagement in tempting, health-compromising behaviors. Future work on this topic could clarify when and why CHBs contribute to health-compromising behavior, and help identify effective strategies for weakening their influence, ultimately improving intervention outcomes and supporting long-term behavior change.

### CHBs and compensatory intentions versus behaviors

As predicted, on average, CHBs were significantly, positively associated with compensatory intentions, but not with actual behavior. This pattern of results suggests that while people may form an intention to engage in a compensatory health behavior to reduce one’s dissonance, these intentions are rarely carried out, reflecting the intention-behavior gap.^20^ Implementation intentions, or if-then plans that pair a contextual cue with behavioral action have previously been shown to be an effective approach to bridging this intention-behavior gap.^70^ Although CHBs (“If I eat a donut, I can compensate for it by going to the gym later”) may resemble implementation intentions (eg, If I eat a donut, then I will go to the gym after work), they constitute the belief that compensation is possible rather than a specific plan to act based on a cue. Indeed, the fact that CHBs often involve vague and/or delayed actions likely contributes to them being ineffective for promoting compensatory health behaviors.^71^^,^^72^ In order to promote follow-through to perform a health-promoting compensatory behavior, people could be encouraged to create specific plans for when, where, and how they will perform a health promoting behavior (eg, “When I finish work, then I will go to the gym”). Such implementation intentions have been shown to promote behavior change even when self-control is depleted,^73^ making them especially relevant given that CHBs often emerge in the context of self-regulation failure, either in anticipation of it or as a response to it.^4^ Future studies should test if helping people create implementation intentions leads to greater engagement in compensatory health behaviors when CHBs are activated compared to when no specific plans are made.

### Moderators of CHB-intention and CHB-behavior relationships

Given the mixed findings in the existing literature, our review and meta-analytic approach sought to identify potential sources of heterogeneity. Contrary to most of our predictions, only the type of behavior studied significantly moderated the relationship between CHBs and a single outcome. Specifically, CHBs were more strongly associated with compensatory intentions in the context of diet than physical activity. This finding may reflect that healthy eating is perceived as a more immediate or accessible compensatory health behavior, whereas physical activity typically requires more time, effort, and planning.^40^ Health-compromising intentions and behaviors were not moderated by behavior type, suggesting that people may be equally willing to abandon diet and physical activity, perhaps in favor of less resource intensive alternatives.^19^ Future research aimed at understanding when CHBs are potentially most harmful, may benefit from comparing behaviors that are categorized in more nuanced ways, including examining characteristics of behaviors such as effort, accessibility, or approach/avoidance.^74^

The lack of moderation effects across other variables (ie, self-efficacy, time, specificity of CHBs, and risk perception) may reflect a lack of consistency in how these constructs are operationalized across studies or the limited number of studies that have directly assessed these moderators. This paper highlights the importance of greater consistency in how researchers operationalize constructs, as well as the need for more research examining factors that could explain when and how CHBs are related to health intentions and behaviors.

### Studying CHBs moving forward

The current meta-analyses highlight both the breadth of research on CHBs and several key gaps in the literature. Most notably, the majority of studies reviewed relied on convenience samples of healthy, college-age individuals. Yet, health behavior change is most consequential, and arguably more likely, among populations for whom the risks of inaction are more severe.^75^ Indeed, prior research on individuals managing chronic illnesses like cardiovascular disease showed that CHBs are more likely to be associated with health behavior change when risk was medium-to-high rather than low.^19^ Research comparing older and clinical, with younger and non-clinical, populations would not only enhance generalizability but also provide critical insight into how CHBs function in higher-stakes contexts. In addition, many studies to date have focused on a narrow range of health behaviors, leaving important domains including, but not limited to, medication adherence, sleep, and dental hygiene underexamined. Broadening the scope of health behaviors studied, and doing so in more diverse populations, will provide a more complete understanding of the relationships among CHBs and behavioral outcomes.

It is also worth mentioning that reliability for CHBs across the studies identified tended to be low, a problem inherent in research on CHBs.^3^^,^^23^ Although our analysis did not reveal any differences in effect sizes as a function of Cronbach’s alpha, our review reinforces calls to develop and use reliable and valid measures of CHBs. For example, researchers have previously called for CHB measures that use first person language, avoid confusing terminology (eg, “compensate for” should be used in place of “make-up for”), as well as frame questions in relation to a specific situation and time period (eg, “I believe that if I exercise tomorrow it will compensate for eating a piece of cake today”).^4^^,^^15^^,^^23^^,^^66^^,^^76^ More research should test whether these improvements to CHB scales enhance predictive validity of behavioral outcomes.

This review also highlights the need for future studies to examine CHBs in the context of multiple health behaviors. In the real world, people rarely make decisions about health behaviors in isolation. Instead, behaviors cluster, compete, and interact;^77^ often drawing on shared resources such as time, motivation, or self-regulatory capacity.^10^^,^^18^^,^^78^ At these intersections, CHBs may emerge as individuals attempt to reconcile competing demands (eg, skipping the gym because they ate a salad earlier),^4^^,^^5^ yet few studies have simultaneously assessed CHBs alongside both the health-compromising and compensatory health behaviors to which they are theorized to be linked.^13^^,^^19^ Studying both compensatory and health-compromising behaviors together, rather than in parallel or in isolation, is crucial for identifying when CHBs reinforce patterns of healthy and/or unhealthy behaviors. Doing so could lead to refinements in theories of multiple health behavior change and help design interventions that better reflect the complexity of everyday life, where changing one behavior often affects others.^10^ Understanding these dynamics will be critical for optimizing health outcomes through interventions that target multiple behaviors simultaneously, thereby saving both time and resources.^79^

### Limitations

This study provides the first meta-analytic synthesis of CHBs across multiple health domains, but several limitations should be noted. First, most studies in this area examine people’s general tendency to endorse CHBs,^15^ and not CHBs in response to specific temptations or health-compromising acts. Indeed, our review of the literature identified only a few studies that assessed CHBs at the moment when a person was faced with a tempting choice.^14^^,^^21^^,^^66–68^^,^^80^ It is possible that the current meta-analysis underestimates the strength of the association between CHBs and behavioral outcomes (especially intentions to perform a health-compromising act) because of the limited studies capturing CHBs at the moment when dissonance is experienced. More research is needed to disentangle the state and trait level effects of CHBs on intentions and behaviors.

While there was a great deal of variability in the synthesized results, very little of this heterogeneity was explained by the anticipated moderators, highlighting the need for more research examining factors that could play a role in these relationships. Further, the moderation analyses were based on subsets of studies where the necessary information was available, but it is not possible to determine if the data was missing at random and the reader should be aware that these results may be biased in some way.^59^ Small sample sizes in these analyses, particularly those reporting on risk perception, led to unstable estimates (*df* < 4), limiting interpretation.^47^^,^^48^^,^^54^

### Conclusion

Unhealthy behaviors do not occur in isolation, but rather, frequently cluster together, resulting in a greater risk of noncommunicable diseases (eg, heart disease, cancer) and mental health issues.^81^ This clustering underscores the need for theoretical frameworks that account for the interplay between health behaviors, and interventions that target multiple health behaviors in an integrated way. CHBs are a type of cross-behavior cognition that offer a promising theoretical lens through which to understand these interdependencies, particularly how individuals navigate trade-offs between behaviors. The findings of the present study inform this work by using a meta-analytical approach to demonstrate that CHBs tend to be positively associated with health-compromising behaviors and compensatory intentions, but not with compensatory health behaviors or intentions to engage in unhealthy actions. The majority of studies reviewed focused on the relationships among CHBs with physical activity and diet behaviors, using correlational studies on primarily college samples, suggesting future work examining the relationships between CHBs and intentions, particularly in the context of other health behaviors, employing experimental methods, and in samples for whom health goals may be particularly relevant (eg, clinical, geriatric) is warranted.

## Supplementary Material

kaag013_Supplementary_Data

## Transparency statements

This study was not formally registered. The analysis plan was not formally pre-registered. De-identified data, all analytic code, and supplementary materials from this study are publicly available on the open science framework (OSF): https://osf.io/5sn93.
