# Supplementary material for: Vices and vegetables: a systematic review and series of meta-analyses examining the relationships among compensatory health beliefs with health-related intentions and behaviors
Source: Ann Behav Med. 2026 Apr 19;60(1):kaag013. doi: 10.1093/abm/kaag013 (PMC13092134; doi:10.1093/abm/kaag013)
Supplement: kaag013_Supplementary_Data [file kaag013_supplementary_data.zip › CHBs Meta-analysis Tables Finalized 3.13.26.docx]

**Supplementary Table 1.**

*Study characteristics for studies included in analyses of CHBs and health-compromising intentions*

|  | | Study Metadata | | | | | | Categorical Moderator Coding | | | Continuous Moderator Coding | | | |
| --- | --- | --- | --- | --- | --- | --- | --- | --- | --- | --- | --- | --- | --- | --- |
| Study ID | Effect Size ID | | Publication Status | Sample | *N* | % Women | Behavior | Design/ Time | General vs Specific CHB | Behavior Category | Alpha of CHB Scale | Self-Efficacy | Risk Perception | Outcome Expectancies |
| 01 (Amrein et al., 2017a) | (Amrein et al., 2017aa) | | PUB | STU | 124 | 76.3 | High-calorie snack consumption | LT | Specific | Diet | — | 1 | 1 | 1 |
| 02 (Amrein et al., 2017b) | (Amrein et al., 2017ba) | | PUB | STU | 107 | 76.3 | Eating little fruit and vegetables | LT | Specific | Diet | — | 1 | 1 | 1 |
| 06 (Duan et al., 2024) | (Duan et al., 2024c) | | UPUB | STU | 322 | 55.6 | Insufficient exercise | LT | Specific | PA | 0.75 | 1 | 0 | 0 |
| 06 (Duan et al., 2024) | (Duan et al., 2024d) | | UPUB | STU | 322 | 55.6 | Insufficient fruit and vegetables | LT | Specific | Diet | 0.85 | 1 | 0 | 0 |
| 07 (Ernsting et al., 2013) | (Ernsting et al., 2013b) | | PUB | GP | 851 | 46.4 | Vaccination | LT | Specific | — | — | 1 | 0 | 0 |
| 09 (Fleig et al., 2015) | (Fleig et al., 2015a) | | PUB | GP | 767 | — | Physical activity | CS | Specific | PA | — | 1 | 1 | 0 |
| 09 (Fleig et al., 2015) | (Fleig et al., 2015b) | | PUB | GP | 767 | — | Diet | CS | Specific | Diet |  | 1 | 1 | 0 |
| 10 (Forestier et al., 2020) | (Forestier et al., 2020a) | | PUB | CLIN | 104 | 19.3 | Physical activity | CS | Specific | PA | — | 1 | 1 | 0 |
| 10 (Forestier et al., 2020) | (Forestier et al., 2020b) | | PUB | CLIN | 104 | 19.3 | Physical activity | CS | Specific | PA | — | 1 | 1 | 0 |
| 10 (Forestier et al., 2020) | (Forestier et al., 2020c) | | PUB | CLIN | 104 | 19.3 | Diet | CS | Specific | Diet | — | 1 | 1 | 0 |
| 10 (Forestier et al., 2020) | (Forestier et al., 2020d) | | PUB | CLIN | 104 | 19.3 | Diet | CS | Specific | Diet | — | 1 | 1 | 0 |
| 11 (Fox, Unpublished Data) | (Fox, n.d.,a) | | UPUB | STU | 251 | 68 | Lack of exercise | CS | Specific | PA | 0.68 | 1 | 0 | 0 |
| 11 (Fox, Unpublished Data) | (Fox, n.d.,b) | | UPUB | STU | 232 | 68 | Lack of exercise | CS | Specific | PA | 0.727 | 1 | 0 | 0 |
| 11 (Fox, Unpublished Data) | (Fox, n.d.,c) | | UPUB | STU | 231 | 68 | Lack of exercise | CS | Specific | PA | 0.796 | 1 | 0 | 0 |
| 11 (Fox, Unpublished Data) | (Fox, n.d.,d) | | UPUB | STU | 213 | 68 | Lack of exercise | LT | Specific | PA | 0.68 | 1 | 0 | 0 |
| 11 (Fox, Unpublished Data) | (Fox, n.d.,e) | | UPUB | STU | 216 | 68 | Lack of exercise | LT | Specific | PA | 0.727 | 1 | 0 | 0 |
| 11 (Fox, Unpublished Data) | (Fox, n.d.,f) | | UPUB | STU | 224 | 68 | Lack of exercise | LT | Specific | PA | 0.68 | 1 | 0 | 0 |
| 11 (Fox, Unpublished Data) | (Fox, n.d.,g) | | UPUB | STU | 251 | 68 | Lack of eating fruits and vegetables | CS | Specific | Diet | 0.657 | 1 | 0 | 0 |
| 11 (Fox, Unpublished Data) | (Fox, n.d.,h) | | UPUB | STU | 232 | 68 | Lack of eating fruits and vegetables | CS | Specific | Diet | 0.743 | 1 | 0 | 0 |
| 11 (Fox, Unpublished Data) | (Fox, n.d.,i) | | UPUB | STU | 231 | 68 | Lack of eating fruits and vegetables | CS | Specific | Diet | 0.768 | 1 | 0 | 0 |
| 11 (Fox, Unpublished Data) | (Fox, n.d.,j) | | UPUB | STU | 213 | 68 | Lack of eating fruits and vegetables | LT | Specific | Diet | 0.657 | 1 | 0 | 0 |
| 11 (Fox, Unpublished Data) | (Fox, n.d.,k) | | UPUB | STU | 216 | 68 | Lack of eating fruits and vegetables | LT | Specific | Diet | 0.743 | 1 | 0 | 0 |
| 11 (Fox, Unpublished Data) | (Fox, n.d.,l) | | UPUB | STU | 224 | 68 | Lack of eating fruits and vegetables | LT | Specific | Diet | 0.657 | 1 | 0 | 0 |
| 17 (Hein, 2014) | (Hein, 2014a) | | UPUB | STU | 113 | 79 | Alcohol consumption | CS | Specific | Alcohol | 0.615 | 1 | 0 | 0 |
| 23 (Kronick & Knäuper, 2010) | (Kronick & Knäuper, 2010) | | PUB | CLIN | 21 | 100 | Eating an unhealthy cookie | CS | General | Diet | — | 1 | 0 | 0 |
| 25 (Kruse, 2015) | (Kruse, 2015) | | UPUB | STU | 105 | 32.4 | Alcohol consumption | CS | Specific | Alcohol | 0.8 | 1 | 0 | 0 |
| 40 (Radtke & Inauen et al., 2014) | (Radtke & Inauen et al., 2014a) | | PUB | STU | 67 | 100 | Diet (snack consumption) | CS | Specific | Diet | 0.77 | 0 | 0 | 0 |
| 41 (Radtke & Kaklamanou et al., 2014) | (Radtke & Kaklamanou et al., 2014) | | PUB | STU | 75 | 100 | Diet (eating without restrictions or in an unhealthy manner) | CS | Specific | Diet | 0.44 | 1 | 1 | 1 |
| 43 (Radtke & Scholz, 2017) | (Radtke & Scholz, 2017b) | | PUB | STU | 227 | 61.7 | Alcohol consumption | CS | Specific | Alcohol | T1: 0.93 | 0 | 0 | 0 |
| 44 (Radtke et al., 2012) | (Radtke et al., 2012) | | PUB | CLIN | 385 | 50.4 | Smoking (Cigarettes per day) | CS | Specific | Smoking | 0.89 | 1 | 1 | 1 |
| 46 (Ricker, 2013) | (Ricker, 2013) | | UPUB | STU | 167 | 81.4 | Binge Drinking alcohol | CS | Specific | Alcohol | 0.88 | 1 | 0 | 0 |
| 52 (Storm et al., 2017) | (Storm et al., 2017) | | PUB | CLIN | 790 | 62.9 | Low fruit and vegetable consumption | CS | Specific | Diet | 0.72 | 1 | 0 | 0 |
| 54 (te Wilde, 2013) | (te Wilde, 2013) | | UPUB | STU | 170 | 81.2 | Snack consumption (Fat list) | CS | Specific | Diet | 0.68 | 1 | 0 | 0 |

*Note.* Study ID = Identifier for independent samples; Effect Size ID = Identifier of a specific correlation between a given CHB and a single behavioral outcome; CHB = Compensatory health belief; PUB = Published; UPUB = Unpublished; STU = Student; GP = General population; CLIN = Clinical; CS = Cross-sectional; LT = Longitudinal; Alpha = Cronbach’s α. For continuous moderator coding, 0 = Absent; 1 = Present

**Supplementary Table 2**

*Study characteristics for studies included in analyses of CHBs and health-compromising behaviors*

|  | Study Metadata | | | | | | Categorical Moderator Coding | | | Continuous Moderator Coding | | | |
| --- | --- | --- | --- | --- | --- | --- | --- | --- | --- | --- | --- | --- | --- |
| Study ID | Effect Size ID | Publication Status | Sample | *N* | % Women | Behavior | Design/ Time | General vs Specific CHB | Behavior Category | Alpha of CHB Scale | Self-Efficacy | Risk perception | Outcome Expectancies |
| 01 (Amrein et al., 2017a) | (Amrein et al., 2017ab) | PUB | STU | 232 | 76.3 | High-calorie snack consumption | LT | Specific | Diet | — | 1 | 1 | 1 |
| 02 (Amrein et al., 2017b) | (Amrein et al., 2017bb) | PUB | STU | 232 | 76.3 | Eating little fruit and vegetables | LT | Specific | Diet | — | 1 | 1 | 1 |
| 03 (An & Zhang, 2024) | (An & Zhang, 2024) | PUB | GP | 999 | 74.87 | Bedtime Procrastination | CS | Specific | — | — | 0 | 0 | 0 |
| 04 (Austermann, 2016) | (Austermann, 2016a) | UPUB | STU | 100 | 69 | Smoking | CS | General | Smoking | 0.76 | 0 | 0 | 0 |
| 04 (Austermann, 2016) | (Austermann, 2016b) | UPUB | STU | 100 | 69 | Smoking | CS | Specific | Smoking | 0.53 | 0 | 0 | 0 |
| 04 (Austermann, 2016) | (Austermann, 2016c) | UPUB | STU | 100 | 69 | Alcohol consumption | CS | General | Alcohol | 0.76 | 0 | 0 | 0 |
| 04 (Austermann, 2016) | (Austermann, 2016d) | UPUB | STU | 100 | 69 | Alcohol consumption | CS | Specific | Alcohol | 0.53 | 0 | 0 | 0 |
| 06 (Duan et al., 2024) | (Duan et al., 2024a) | UPUB | STU | 322 | 55.6 | Insufficient physical activity | LT | Specific | PA | 0.75 | 1 | 0 | 0 |
| 06 (Duan et al., 2024) | (Duan et al., 2024b) | UPUB | STU | 322 | 55.6 | Insufficient fruit and vegetable consumption | LT | Specific | Diet | 0.85 | 1 | 0 | 0 |
| 06 (Duan et al., 2024) | (Duan et al., 2024c) | UPUB | STU | 322 | 55.6 | Insufficient physical activity | CS | Specific | PA | 0.75 | 1 | 0 | 0 |
| 06 (Duan et al., 2024) | (Duan et al., 2024d) | UPUB | STU | 322 | 55.6 | Insufficient fruit and vegetable consumption | CS | Specific | Diet | 0.85 | 1 | 0 | 0 |
| 07 (Ernsting et al., 2013) | (Ernsting et al., 2013a) | PUB | GP | 851 | 46.4 | Vaccination | CS | Specific | — | — | 1 | 0 | 0 |
| 07 (Ernsting et al., 2013) | (Ernsting et al., 2013b) | PUB | GP | 851 | 46.4 | Vaccination | LT | Specific | — | — | 1 | 0 | 0 |
| 08 (Finlay et al., 2024) | (Finlay et al., 2024a) | PUB | GP | 34 | 61.8 | Energy consumption (of unhealthy desserts) | CS | Specific | Diet | — | 0 | 0 | 0 |
| 08 (Finlay et al., 2024) | (Finlay et al., 2024b) | PUB | GP | 32 | 65.6 | Energy consumption (lunch, dinner, snacks, and dessert) | CS | Specific | Diet | — | 0 | 0 | 0 |
| 09 (Fleig et al., 2015) | (Fleig et al., 2015a) | PUB | GP | 767 | — | Physical activity | CS | Specific | PA | — | 1 | 1 | 0 |
| 09 (Fleig et al., 2015) | (Fleig et al., 2015b) | PUB | GP | 767 | — | Diet | CS | Specific | Diet | — | 1 | 1 | 0 |
| 11 (Fox, Unpublished Data) | (Fox, n.d.,a) | UPUB | STU | 251 | 68 | Lack of exercise | CS | Specific | PA | — | 0 | 0 | 0 |
| 11 (Fox, Unpublished Data) | (Fox, n.d.,b) | UPUB | STU | 232 | 68 | Lack of exercise | CS | Specific | PA | — | 0 | 0 | 0 |
| 11 (Fox, Unpublished Data) | (Fox, n.d.,c) | UPUB | STU | 231 | 68 | Lack of exercise | CS | Specific | PA | — | 0 | 0 | 0 |
| 11 (Fox, Unpublished Data) | (Fox, n.d.,d) | UPUB | STU | 213 | 68 | Lack of exercise | LT | Specific | PA | — | 0 | 0 | 0 |
| 11 (Fox, Unpublished Data) | (Fox, n.d.,e) | UPUB | STU | 216 | 68 | Lack of exercise | LT | Specific | PA | — | 0 | 0 | 0 |
| 11 (Fox, Unpublished Data) | (Fox, n.d.,f) | UPUB | STU | 224 | 68 | Lack of exercise | LT | Specific | PA | — | 0 | 0 | 0 |
| 11 (Fox, Unpublished Data) | (Fox, n.d.,g) | UPUB | STU | 251 | 68 | Lack of eating fruits and vegetables | CS | Specific | Diet | — | 0 | 0 | 0 |
| 11 (Fox, Unpublished Data) | (Fox, n.d.,h) | UPUB | STU | 232 | 68 | Lack of eating fruits and vegetables | CS | Specific | Diet | — | 0 | 0 | 0 |
| 11 (Fox, Unpublished Data) | (Fox, n.d.,i) | UPUB | STU | 231 | 68 | Lack of eating fruits and vegetables | CS | Specific | Diet | — | 0 | 0 | 0 |
| 11 (Fox, Unpublished Data) | (Fox, n.d.,j) | UPUB | STU | 213 | 68 | Lack of eating fruits and vegetables | LT | Specific | Diet | — | 0 | 0 | 0 |
| 11 (Fox, Unpublished Data) | (Fox, n.d.,k) | UPUB | STU | 216 | 68 | Lack of eating fruits and vegetables | LT | Specific | Diet | — | 0 | 0 | 0 |
| 11 (Fox, Unpublished Data) | (Fox, n.d.,l) | UPUB | STU | 224 | 68 | Lack of eating fruits and vegetables | LT | Specific | Diet | — | 0 | 0 | 0 |
| 12 (Gallagher, 2019) | (Gallagher, 2019a) | PUB | STU | 101 | 64 | Poor diet | CS | Specific | Diet | 0.82 | 0 | 0 | 0 |
| 12 (Gallagher, 2019) | (Gallagher, 2019b) | PUB | STU | 101 | 64 | Poor diet | CS | General | Diet | — | 0 | 0 | 0 |
| 13 (Glock et al., 2013a) | (Glock et al., 2013a) | PUB | STU | 49 | 46.7 | Smoking (Cigarettes per day) | CS | Specific | Smoking | — | 0 | 0 | 0 |
| 15 (Gough et al., 2025) | (Gough et al., 2025a) | PUB | GP | 36 | 50 | Eating more | CS | Specific | Diet | 0.81 | 0 | 0 | 0 |
| 15 (Gough et al., 2025) | (Gough et al., 2025b) | PUB | GP | 36 | 50 | Eating more | LT | Specific | Diet | 0.81 | 0 | 0 | 0 |
| 17 (Hein, 2014) | (Hein, 2014a) | UPUB | STU | 113 | 79 | Alcohol consumption | CS | Specific | Alcohol | 0.615 | 1 | 0 | 0 |
| 17 (Hein, 2014) | (Hein, 2014b) | UPUB | STU | 113 | 79 | Binge drinking | CS | Specific | Alcohol | 0.615 | 1 | 0 | 0 |
| 19 (Hoffman, 2023) | (Hoffman, 2023a) | UPUB | STU | 200 | 100 | Calories from alcohol consumption | CS | Specific | Alcohol | 0.91 | 1 | 0 | 0 |
| 19 (Hoffman, 2023) | (Hoffman, 2023b) | UPUB | STU | 200 | 100 | Calories from alcohol consumption | CS | Specific | Alcohol | 0.92 | 1 | 0 | 0 |
| 19 (Hoffman, 2023) | (Hoffman, 2023c) | UPUB | STU | 200 | 100 | Calories from alcohol consumption | CS | Specific | Alcohol | 0.92 | 1 | 0 | 0 |
| 19 (Hoffman, 2023) | (Hoffman, 2023d) | UPUB | STU | 200 | 100 | Calories from alcohol consumption | CS | Specific | Alcohol | 0.81 | 1 | 0 | 0 |
| 20 (Kaklamanou & Armitage, 2012) | (Kaklamanou & Armitage, 2012a) | PUB | GP | 263 | 75.7 | Exercise (Moderate activity) | CS | Specific | PA | 0.72 | 0 | 0 | 0 |
| 22 (Knäuper et al., 2004) | (Knäuper et al., 2004a) | PUB | STU | 111 | 75.4 | General health risk behaviors | CS | General | — | 0.76 | 3 | 0 | 0 |
| 22 (Knäuper et al., 2004) | (Knäuper et al., 2004b) | PUB | STU | 111 | 48.6 | Alcohol/nicotine related risk behavior | CS | Specific | Smoking | 0.74 | 1 | 0 | 0 |
| 22 (Knäuper et al., 2004) | (Knäuper et al., 2004c) | PUB | STU | 111 | 48.6 | Eating-related risk behavior | CS | Specific | Diet | 0.66 | 1 | 0 | 0 |
| 22 (Knäuper et al., 2004) | (Knäuper et al., 2004d) | PUB | STU | 111 | 48.6 | Weight regulation risk behavior | CS | Specific | Diet | 0.57 | 1 | 0 | 0 |
| 23 (Kronick & Knäuper, 2010) | (Kronick & Knäuper, 2010) | PUB | CLIN | 21 | 100 | Eating a high calorie cookie | CS | General | Diet | — | 1 | 0 | 0 |
| 24 (Kronick et al., 2011) | (Kronick et al., 2011) | PUB | STU | 69 | 87 | Caloric intake | LT | General | Diet | 0.84 | 0 | 0 | 0 |
| 27 (Matley & Davies, 2018) | (Matley & Davies, 2018) | PUB | GP | 249 | 63.1 | Alcohol consumption | CS | Specific | Alcohol | 0.787 | 0 | 0 | 0 |
| 29 (Miquelon et al., 2012) | (Miquelon et al., 2012) | PUB | CLIN | 119 | 100 | Diet non-adherence or unhealthy behavior choices toward weight regulation | LT | Specific | Diet | 0.68 | 0 | 0 | 0 |
| 30 (Moll, 2014) | (Moll, 2014) | UPUB | STU | 68 | 82.4 | Tanning | CS | Specific | — | 0.915 | 0 | 0 | 0 |
| 31 (Nasser, 2016) | (Nasser, 2016a) | UPUB | GP | 88 | 68.2 | Binge eating | CS | Specific | Diet | 0.71 | 0 | 0 | 0 |
| 31 (Nasser, 2016) | (Nasser, 2016b) | UPUB | GP | 88 | 68.2 | Binge eating | CS | Specific | Diet | 0.54 | 0 | 0 | 0 |
| 32 (Natrop, 2015) | (Natrop, 2015b) | UPUB | STU | 139 | 64 | Snack consumption | CS | Specific | Diet | 0.85 | 0 | 0 | 0 |
| 33 (Neufeld, 2015) | (Neufeld, 2015a) | UPUB | STU | 139 | 64 | Physical inactivity | CS | Specific | PA | 0.66 | 0 | 0 | 0 |
| 33 (Neufeld, 2015) | (Neufeld, 2015b) | UPUB | STU | 139 | 64 | Physical inactivity | CS | Specific | PA | 0.66 | 0 | 0 | 0 |
| 33 (Neufeld, 2015) | (Neufeld, 2015c) | UPUB | STU | 139 | 64 | Physical inactivity | CS | Specific | PA | 0.66 | 0 | 0 | 0 |
| 34 (Oberschmidt, 2017) | (Oberschmidt, 2017a) | UPUB | STU | 320 | 72 | Binge-watching frequency | CS | Specific | PA | 0.83 | 0 | 0 | 0 |
| 34 (Oberschmidt, 2017) | (Oberschmidt, 2017b) | UPUB | STU | 320 | 72 | Binge-watching frequency | CS | General | PA | 0.78 | 0 | 0 | 0 |
| 35 (Olding, 2018) | (Olding, 2018a) | UPUB | STU | 172 | 81.4 | Binge-watching quantity | CS | Specific | PA | 0.82 | 1 | 0 | 0 |
| 35 (Olding, 2018) | (Olding, 2018b) | UPUB | STU | 172 | 81.4 | Binge-watching frequency | CS | Specific | PA | 0.82 | 1 | 0 | 0 |
| 36 (Paulus & Aziz, 2023) | (Paulus & Aziz, 2023) | PUB | STU | 167 | 66.5 | Binge watching | CS | Specific | PA | 0.69 | 0 | 0 | 0 |
| 37 (Pink et al., 2022) | (Pink et al., 2022) | PUB | STU | 148 | 54.7 | Unhealthy snack (Chip intake) | CS | General | Diet | 0.8 | 0 | 0 | 0 |
| 38 (Prinsen, 2017) | (Prinsen, 2017a) | UPUB | STU | 330 | 72.7 | Binge-watching | CS | General | PA | 0.88 | 0 | 0 | 0 |
| 38 (Prinsen, 2017) | (Prinsen, 2017b) | UPUB | STU | 330 | 72.7 | Binge-watching | CS | Specific | PA | 0.78 | 0 | 0 | 0 |
| 39 (Rabiau et al., 2009) | (Rabiau et al., 2009) | PUB | CLIN | 114 | 56.1 | Glucose-testing frequency | CS | Specific | — | 0.81 | 1 | 0 | 0 |
| 40 (Radtke & Inauen et al., 2014) | (Radtke & Inauen et al., 2014a) | PUB | STU | 67 | 100 | Diet (Snack consumption) | CS | Specific | Diet | 0.77 | 0 | 0 | 0 |
| 40 (Radtke & Inauen et al., 2014) | (Radtke & Inauen et al., 2014b) | PUB | STU | 67 | 100 | Diet (Snack consumption) | CS | Specific | Diet | 0.71 | 0 | 0 | 0 |
| 41 (Radtke & Kaklamanou et al., 2014) | (Radtke & Kaklamanou et al., 2014) | PUB | STU | 75 | 100 | Diet (Eating without restrictions or unhealthy eating) | CS | Specific | Diet | 0.44 | 1 | 1 | 1 |
| 42 (Radtke & Rackow, 2014) | (Radtke & Rackow, 2014) | PUB | STU | 135 | 51.5 | Physical inactivity (Sedentary behavior like elevator use) | CS | Specific | PA | 0.84 | 0 | 0 | 0 |
| 43 (Radtke & Scholz, 2017) | (Radtke & Scholz, 2017a) | PUB | STU | 227 | 61.7 | Alcohol consumption | LT | Specific | Alcohol | 0.93 | 0 | 0 | 0 |
| 43 (Radtke & Scholz, 2017) | (Radtke & Scholz, 2017b) | PUB | STU | 227 | 61.7 | Alcohol consumption | CS | Specific | Alcohol | 0.93 | 0 | 0 | 0 |
| 44 (Radtke et al., 2012) | (Radtke et al., 2012) | PUB | CLIN | 385 | 50.4 | Smoking (Cigarettes per day) | LT | Specific | Smoking | 0.89 | 1 | 1 | 1 |
| 45 (Ramirez, 2017) | (Ramirez, 2017a) | UPUB | STU | 222 | 45.5 | Frequency of engaging in PBS (Stopping/limiting drinking) | CS | Specific | Alcohol | 0.915 | 1 | 0 | 1 |
| 45 (Ramirez, 2017) | (Ramirez, 2017b) | UPUB | STU | 222 | 45.5 | Frequency of engaging in PBS (Manner of drinking) | CS | Specific | Alcohol | 0.915 | 1 | 0 | 1 |
| 45 (Ramirez, 2017) | (Ramirez, 2017c) | UPUB | STU | 222 | 45.5 | Frequency of engaging in PBS (Serious harm reduction) | CS | Specific | Alcohol | 0.915 | 1 | 0 | 1 |
| 45 (Ramirez, 2017) | (Ramirez, 2017d) | UPUB | STU | 222 | 45.5 | Frequency of engaging in PBS (Stopping/limiting drinking) | CS | Specific | Alcohol | 0.789 | 1 | 0 | 1 |
| 45 (Ramirez, 2017) | (Ramirez, 2017e) | UPUB | STU | 222 | 45.5 | Frequency of engaging in PBS (Manner of drinking) | CS | Specific | Alcohol | 0.789 | 1 | 0 | 1 |
| 45 (Ramirez, 2017) | (Ramirez, 2017f) | UPUB | STU | 222 | 45.5 | Frequency of engaging in PBS (Serious harm reduction) | CS | Specific | Alcohol | 0.789 | 1 | 0 | 1 |
| 46 (Ricker, 2013) | (Ricker, 2013) | UPUB | STU | 167 | 81.4 | Binge drinking alcohol | CS | Specific | Alcohol | 0.88 | 1 | 0 | 0 |
| 47 (Scheffels, 2016) | (Scheffels, 2016a) | UPUB | STU | 209 | 63 | Alcohol consumption | CS | Specific | Alcohol | 0.73 | 0 | 0 | 0 |
| 47 (Scheffels, 2016) | (Scheffels, 2016b) | UPUB | STU | 209 | 63 | Alcohol consumption | CS | General | Alcohol | 0.79 | 0 | 0 | 0 |
| 47 (Scheffels, 2016) | (Scheffels, 2016c) | UPUB | STU | 209 | 63 | Binge drinking | CS | Specific | Alcohol | 0.73 | 0 | 0 | 0 |
| 47 (Scheffels, 2016) | (Scheffels, 2016d) | UPUB | STU | 209 | 63 | Binge drinking | CS | General | Alcohol | 0.79 | 0 | 0 | 0 |
| 49 (Sim & Cheon, 2019) | (Sim & Cheon, 2019) | PUB | STU | 51 | 60.6 | Unhealthy snacking | CS | Specific | Diet | 0.76 | 0 | 0 | 0 |
| 50 (Smoletz, 2016) | (Smoletz, 2016a) | UPUB | STU | 195 | 58.5 | Unhealthy eating | CS | General | Diet | 0.7 | 0 | 0 | 0 |
| 50 (Smoletz, 2016) | (Smoletz, 2016b) | UPUB | STU | 195 | 58.5 | Unhealthy eating | CS | Specific | Diet | 0.81 | 0 | 0 | 0 |
| 53 (Tăut & Băban, 2008) | (Tăut & Băban, 2008) | PUB | CLIN | 55 | 50.9 | Unhealthy eating | CS | Specific | Diet | — | 1 | 0 | 0 |
| 54 (te Wilde, 2013) | (te Wilde, 2013) | UPUB | STU | 170 | 81.2 | Snack consumption (fat list) | CS | Specific | Diet | 0.66 | 1 | 0 | 0 |
| 55 (Thomas, 2016) | (Thomas, 2016a) | UPUB | STU | 216 | 62.5 | Alcohol consumption during weekday | CS | Specific | Alcohol | 0.8 | 1 | 0 | 0 |
| 55 (Thomas, 2016) | (Thomas, 2016b) | UPUB | STU | 216 | 62.5 | Alcohol consumption during weekend | CS | Specific | Alcohol | 0.8 | 1 | 0 | 0 |
| 55 (Thomas, 2016) | (Thomas, 2016c) | UPUB | STU | 216 | 62.5 | Alcohol consumption during weekday | CS | Specific | Alcohol | 0.79 | 1 | 0 | 0 |
| 55 (Thomas, 2016) | (Thomas, 2016d) | UPUB | STU | 216 | 62.5 | Alcohol consumption during weekend | CS | Specific | Alcohol | 0.79 | 1 | 0 | 0 |
| 58 (Zhou et al., 2016) | (Zhou et al., 2016a) | PUB | GP | 140 | 40 | Make a call while driving | CS | General | — | 0.91 | 0 | 0 | 0 |
| 58 (Zhou et al., 2016) | (Zhou et al., 2016b) | PUB | GP | 140 | 40 | Answer a call while driving | CS | General | — | 0.91 | 0 | 0 | 0 |
| 58 (Zhou et al., 2016) | (Zhou et al., 2016j) | PUB | GP | 140 | 40 | Send a short message | CS | General | — | 0.91 | 0 | 0 | 0 |
| 58 (Zhou et al., 2016) | (Zhou et al., 2016k) | PUB | GP | 140 | 40 | Read a short message | CS | General | — | 0.91 | 0 | 0 | 0 |
| 58 (Zhou et al., 2016) | (Zhou et al., 2016pa) | PUB | GP | 140 | 40 | Make a call while driving | CS | Specific | — | 0.58 | 0 | 0 | 0 |
| 58 (Zhou et al., 2016) | (Zhou et al., 2016pb) | PUB | GP | 140 | 40 | Answer a call while driving | CS | Specific | — | 0.58 | 0 | 0 | 0 |
| 58 (Zhou et al., 2016) | (Zhou et al., 2016pj) | PUB | GP | 140 | 40 | Send a short message | CS | Specific | — | 0.58 | 0 | 0 | 0 |
| 58 (Zhou et al., 2016) | (Zhou et al., 2016pk) | PUB | GP | 140 | 40 | Read a short message | CS | Specific | — | 0.58 | 0 | 0 | 0 |
| 59 (Zhou et al., 2020) | (Zhou et al., 2020a) | PUB | GP | 304 | 38.8 | Mobile phone use | CS | Specific | — | 0.9 | 0 | 1 | 0 |
| 59 (Zhou et al., 2020) | (Zhou et al., 2020b) | PUB | GP | 304 | 38.8 | In vehicle interactions | CS | Specific | — | 0.9 | 0 | 1 | 0 |
| 59 (Zhou et al., 2020) | (Zhou et al., 2020c) | PUB | GP | 304 | 38.8 | Mind wandering/ daydreaming | CS | Specific | — | 0.9 | 0 | 1 | 0 |

*Note.* Study ID = Identifier for independent samples; Effect Size ID = Identifier of a specific correlation between a given CHB and a single behavioral outcome; CHB = Compensatory health belief; PUB = Published; UPUB = Unpublished; STU = Student; GP = General population; CLIN = Clinical; CS = Cross-sectional; LT = Longitudinal; Alpha = Cronbach’s α. For continuous moderator coding, 0 = Absent; 1 = Present

**Supplementary Table 3.**

*Study characteristics for studies included in analyses of CHBs and compensatory intentions*

|  | Study Metadata | | | | | | Categorical Moderator Coding | | | Continuous Moderator Coding | | | |
| --- | --- | --- | --- | --- | --- | --- | --- | --- | --- | --- | --- | --- | --- |
| Study ID | Effect Size ID | Publication Status | Sample | *N* | % Women | Behavior | Design/ Time | General vs Specific CHB | Behavior Category | Alpha of CHB Scale | Self-efficacy | Risk Perception | Outcome Expectancies |
| 05 (Berli et al., 2014) | (Berli et al., 2014a) | PUB | STU* | 430 | 46.3 | Physical activity | CS | General | PA | 0.77 | 1 | 1 | 1 |
| 05 (Berli et al., 2014) | (Berli et al., 2014b) | PUB | STU* | 430 | 46.3 | Physical activity | LT | General | PA | 0.77 | 1 | 1 | 1 |
| 06 (Duan et al., 2024) | (Duan et al., 2024c) | UPUB | STU | 322 | 55.6 | Sufficient fruit and vegetable consumption | LT | Specific | Diet | 0.75 | 1 | 0 | 0 |
| 06 (Duan et al., 2024) | (Duan et al., 2024d) | UPUB | STU | 322 | 55.6 | Sufficient physical activity | LT | Specific | Diet | 0.85 | 1 | 0 | 0 |
| 11 (Fox, Unpublished Data) | (Fox, n.d.,a) | UPUB | STU | 251 | 68 | Eating fruits and vegetables | CS | Specific | Diet | 0.68 | 0 | 0 | 0 |
| 11 (Fox, Unpublished Data) | (Fox, n.d.,b) | UPUB | STU | 232 | 68 | Eating fruits and vegetables | CS | Specific | Diet | 0.727 | 0 | 0 | 0 |
| 11 (Fox, Unpublished Data) | (Fox, n.d.,c) | UPUB | STU | 231 | 68 | Eating fruits and vegetables | CS | Specific | Diet | 0.796 | 0 | 0 | 0 |
| 11 (Fox, Unpublished Data) | (Fox, n.d.,d) | UPUB | STU | 213 | 68 | Eating fruits and vegetables | LT | Specific | Diet | 0.68 | 0 | 0 | 0 |
| 11 (Fox, Unpublished Data) | (Fox, n.d.,e) | UPUB | STU | 216 | 68 | Eating fruits and vegetables | LT | Specific | Diet | 0.727 | 0 | 0 | 0 |
| 11 (Fox, Unpublished Data) | (Fox, n.d.,f) | UPUB | STU | 224 | 68 | Eating fruits and vegetables | LT | Specific | Diet | 0.68 | 0 | 0 | 0 |
| 11 (Fox, Unpublished Data) | (Fox, n.d.,g) | UPUB | STU | 251 | 68 | Exercise | CS | Specific | PA | 0.657 | 0 | 0 | 0 |
| 11 (Fox, Unpublished Data) | (Fox, n.d.,h) | UPUB | STU | 232 | 68 | Exercise | CS | Specific | PA | 0.743 | 0 | 0 | 0 |
| 11 (Fox, Unpublished Data) | (Fox, n.d.,i) | UPUB | STU | 231 | 68 | Exercise | CS | Specific | PA | 0.768 | 0 | 0 | 0 |
| 11 (Fox, Unpublished Data) | (Fox, n.d.,j) | UPUB | STU | 213 | 68 | Exercise | LT | Specific | PA | 0.657 | 0 | 0 | 0 |
| 11 (Fox, Unpublished Data) | (Fox, n.d.,k) | UPUB | STU | 216 | 68 | Exercise | LT | Specific | PA | 0.743 | 0 | 0 | 0 |
| 11 (Fox, Unpublished Data) | (Fox, n.d.,l) | UPUB | STU | 224 | 68 | Exercise | LT | Specific | PA | 0.657 | 0 | 0 | 0 |
| 18 (Heinz, 2013) | (Heinz, 2013a) | UPUB | STU | 242 | 77 | Moderate exercise | CS | General | PA | 0.73 | 1 | 0 | 0 |
| 19 (Hoffman, 2023) | (Hoffman, 2023a) | UPUB | STU | 200 | 100 | Diet | CS | Specific | Diet | 0.91 | 1 | 0 | 0 |
| 19 (Hoffman, 2023) | (Hoffman, 2023b) | UPUB | STU | 200 | 100 | Exercise | CS | Specific | PA | 0.92 | 1 | 0 | 0 |
| 19 (Hoffman, 2023) | (Hoffman, 2023c) | UPUB | STU | 200 | 100 | Drinking less | CS | Specific | — | 0.92 | 1 | 0 | 0 |
| 19 (Hoffman, 2023) | (Hoffman, 2023d) | UPUB | STU | 200 | 100 | Fewer drink calories | CS | Specific | — | 0.81 | 1 | 0 | 0 |
| 23 (Kronick & Knäuper, 2010) | (Kronick & Knäuper, 2010) | PUB | CLIN | 21 | 100 | Diet | CS | General | Diet | — | 1 | 0 | 0 |
| 24 (Kronick et al., 2011) | (Kronick et al., 2011) | PUB | STU | 69 | 87 | Eat better at next meal or exercise to compensate | LT | General | — | 0.84 | 0 | 0 | 0 |
| 26 (Lavins, 2013) | (Lavins, 2013) | UPUB | STU | 212 | 51.9 | Health Behavior Checklist | CS | General | — | 0.752 | 0 | 1 | 0 |
| 28 (Merillat & Gonzalez-Vallejo, 2019) | (Merillat & Gonzalez-Vallejo, 2019) | PUB | GP | 217 | 69.6 | Health Behavior Checklist | CS | General | — | 0.8 | 0 | 1 | 0 |
| 31 (Nasser, 2016) | (Nasser, 2016a) | UPUB | GP | 88 | 68.2 | Exercise later or eat less/more healthily | CS | Specific | — | 0.71 | 0 | 0 | 0 |
| 31 (Nasser, 2016) | (Nasser, 2016b) | UPUB | GP | 88 | 68.2 | Exercise later or eat less/more healthily | CS | Specific | — | 0.54 | 0 | 0 | 0 |
| 37 (Pink et al., 2022) | (Pink et al., 2022) | PUB | STU | 148 | 54.7 | Physical activity | CS | General | PA | 0.8 | 0 | 0 | 0 |
| 40 (Radtke & Inauen et al., 2014) | (Radtke & Inauen et al., 2014b) | PUB | STU | 67 | 100 | Reduced caloric intake | CS | Specific | Diet | 0.71 | 0 | 0 | 0 |
| 41 (Radtke & Kaklamanou et al., 2014) | (Radtke & Kaklamanou et al., 2014) | PUB | STU | 75 | 100 | Diet | CS | Specific | Diet | 0.44 | 1 | 1 | 1 |
| 48 (Selten, 2012) | (Selten, 2012) | UPUB | GP | 597 | 100 | Exercise more and eat less | CS | Specific | PA | 0.58 | 1 | 0 | 0 |
| 51 (Spranger, 2014) | (Spranger, 2014a) | UPUB | STU | 123 | 65.3 | Diet | LT | Specific | Diet | 0.78 | 0 | 0 | 0 |
| 51 (Spranger, 2014) | (Spranger, 2014b) | UPUB | STU | 112 | 65.3 | Diet | LT | Specific | Diet | 0.78 | 0 | 0 | 0 |
| 51 (Spranger, 2014) | (Spranger, 2014c) | UPUB | STU | 100 | 65.3 | Diet | LT | Specific | Diet | 0.81 | 0 | 0 | 0 |
| 56 (Thongworn & Sirisuk, 2018) | (Thongworn & Sirisuk, 2018) | PUB | STU | 788 | 77.7 | Implementation of weight-control behavior | CS | Specific | — | 0.88 | 1 | 0 | 0 |

*Note.* Study ID = Identifier for independent samples; Effect Size ID = Identifier of a specific correlation between a given CHB and a single behavioral outcome; CHB = Compensatory health belief; PUB = Published; UPUB = Unpublished; STU = Student; GP = General population; CLIN = Clinical; CS = Cross-sectional; LT = Longitudinal; Alpha = Cronbach’s α. For continuous moderator coding, 0 = Absent; 1 = Present

*Indicates that the student sample was 8^th^-9^th^ grade students rather than college students which make up the rest of the student sample.

**Supplementary Table 4**

*Study characteristics for studies included in analyses of CHBs and compensatory health behaviors*

|  | | Study Metadata | | | | | | Categorical Moderator Coding | | | Continuous Moderator Coding | | | |
| --- | --- | --- | --- | --- | --- | --- | --- | --- | --- | --- | --- | --- | --- | --- |
| Study ID | Effect Size ID | | Publication Status | Sample | *N* | % Women | Behavior | Design/ Time | General vs Specific CHB | Behavior Category | Alpha of CHB Scale | Self-Efficacy | Risk Perception | Outcome Expectancies |
| 05 (Berli et al., 2014) | (Berli et al., 2014a) | | PUB | STU* | 430 | 46.3 | Physical activity | CS | General | PA | 0.77 | 1 | 1 | 1 |
| 05 (Berli et al., 2014) | (Berli et al., 2014b) | | PUB | STU* | 430 | 46.3 | Physical activity | LT | General | PA | 0.77 | 1 | 1 | 1 |
| 06 (Duan et al., 2024) | (Duan et al., 2024a) | | UPUB | STU | 322 | 55.6 | Sufficient fruit and vegetable consumption | LT | Specific | Diet | 0.75 | 1 | 0 | 0 |
| 06 (Duan et al., 2024) | (Duan et al., 2024b) | | UPUB | STU | 322 | 55.6 | Sufficient physical activity | LT | Specific | PA | 0.85 | 1 | 0 | 0 |
| 06 (Duan et al., 2024) | (Duan et al., 2024c) | | UPUB | STU | 322 | 55.6 | Sufficient fruit and vegetable consumption | CS | Specific | Diet | 0.75 | 1 | 0 | 0 |
| 06 (Duan et al., 2024) | (Duan et al., 2024d) | | UPUB | STU | 322 | 55.6 | Sufficient physical activity | CS | Specific | PA | 0.85 | 1 | 0 | 0 |
| 11 (Fox, Unpublished Data) | (Fox, n.d.,a) | | UPUB | STU | 251 | 68 | Eating fruits and vegetables | CS | Specific | Diet | 0.68 | 0 | 0 | 0 |
| 11 (Fox, Unpublished Data) | (Fox, n.d.,b) | | UPUB | STU | 232 | 68 | Eating fruits and vegetables | CS | Specific | Diet | 0.727 | 0 | 0 | 0 |
| 11 (Fox, Unpublished Data) | (Fox, n.d.,c) | | UPUB | STU | 231 | 68 | Eating fruits and vegetables | CS | Specific | Diet | 0.796 | 0 | 0 | 0 |
| 11 (Fox, Unpublished Data) | (Fox, n.d.,d) | | UPUB | STU | 213 | 68 | Eating fruits and vegetables | LT | Specific | Diet | 0.68 | 0 | 0 | 0 |
| 11 (Fox, Unpublished Data) | (Fox, n.d.,e) | | UPUB | STU | 216 | 68 | Eating fruits and vegetables | LT | Specific | Diet | 0.727 | 0 | 0 | 0 |
| 11 (Fox, Unpublished Data) | (Fox, n.d.,f) | | UPUB | STU | 224 | 68 | Eating fruits and vegetables | LT | Specific | Diet | 0.68 | 0 | 0 | 0 |
| 11 (Fox, Unpublished Data) | (Fox, n.d.,g) | | UPUB | STU | 251 | 68 | Exercise | CS | Specific | PA | 0.657 | 0 | 0 | 0 |
| 11 (Fox, Unpublished Data) | (Fox, n.d.,h) | | UPUB | STU | 232 | 68 | Exercise | CS | Specific | PA | 0.743 | 0 | 0 | 0 |
| 11 (Fox, Unpublished Data) | (Fox, n.d.,i) | | UPUB | STU | 231 | 68 | Exercise | CS | Specific | PA | 0.768 | 0 | 0 | 0 |
| 11 (Fox, Unpublished Data) | (Fox, n.d.,j) | | UPUB | STU | 213 | 68 | Exercise | LT | Specific | PA | 0.657 | 0 | 0 | 0 |
| 11 (Fox, Unpublished Data) | (Fox, n.d.,k) | | UPUB | STU | 216 | 68 | Exercise | LT | Specific | PA | 0.743 | 0 | 0 | 0 |
| 11 (Fox, Unpublished Data) | (Fox, n.d.,l) | | UPUB | STU | 224 | 68 | Exercise | LT | Specific | PA | 0.657 | 0 | 0 | 0 |
| 12 (Gallagher, 2019) | (Gallagher, 2019a) | | PUB | STU | 101 | 64 | Diet and exercise | CS | Specific | — | 0.82 | 0 | 0 | 0 |
| 12 (Gallagher, 2019) | (Gallagher, 2019b) | | PUB | STU | 101 | 64 | Overall eating (Healthy eating vital sign scale) | CS | General | Diet | — | 0 | 0 | 0 |
| 13 (Glock et al., 2013a) | (Glock et al., 2013a) | | PUB | STU | 49 | 46.7 | Choosing a healthy reward (Fruits and cereal bars containing fruits) | CS | Specific | Diet | — | 0 | 0 | 0 |
| 14 (Glock et al., 2013b) | (Glock et al., 2013b) | | PUB | STU | 23 | 46.7 | Choosing a healthy reward (Fruits and cereal bars containing fruits) | CS | General | Diet | — | 0 | 0 | 0 |
| 16 (Hartmann et al., 2016) | (Hartmann et al., 2016a) | | PUB | GP | 490 | 50 | Use of meal replacement products | CS | Specific | Diet | 0.82 | 0 | 0 | 0 |
| 16 (Hartmann et al., 2016) | (Hartmann et al., 2016b) | | PUB | GP | 490 | 50 | Use of meal replacement products | CS | Specific | Diet | 0.76 | 0 | 0 | 0 |
| 17 (Hein, 2014) | (Hein, 2014a) | | UPUB | STU | 113 | 79 | Healthy lifestyle | CS | Specific | — | 0.615 | 1 | 0 | 0 |
| 18 (Heinz, 2013) | (Heinz, 2013a) | | UPUB | STU | 242 | 77 | Moderate exercise | CS | General | PA | 0.73 | 1 | 0 | 0 |
| 18 (Heinz, 2013) | (Heinz, 2013b) | | UPUB | STU | 242 | 77 | Intensive exercise | CS | General | PA | 0.73 | 1 | 0 | 0 |
| 19 (Hoffman, 2023) | (Hoffman, 2023a) | | UPUB | STU | 200 | 100 | Ate less than usual, exercised more than usual, drank less than usual at prior events | CS | Specific | — | 0.91 | 1 | 0 | 0 |
| 19 (Hoffman, 2023) | (Hoffman, 2023b) | | UPUB | STU | 200 | 100 | Ate less than usual, exercised more than usual, drank less than usual at prior events | CS | Specific | — | 0.92 | 1 | 0 | 0 |
| 19 (Hoffman, 2023) | (Hoffman, 2023c) | | UPUB | STU | 200 | 100 | Ate less than usual, exercised more than usual, drank less than usual at prior events | CS | Specific | — | 0.92 | 1 | 0 | 0 |
| 19 (Hoffman, 2023) | (Hoffman, 2023d) | | UPUB | STU | 200 | 100 | Ate less than usual, exercised more than usual, drank less than usual at prior events | CS | Specific | — | 0.81 | 1 | 0 | 0 |
| 20 (Kaklamanou & Armitage, 2012) | (Kaklamanou & Armitage, 2012b) | | PUB | GP | 134 | 75.4 | Exercise (Walking) | LT | Specific | PA | 0.72 | 0 | 0 | 0 |
| 21 (Klein, 2017) | (Klein, 2017) | | UPUB | STU | 331 | 27.8 | Physical activity | CS | General | PA | 0.78 | 1 | 0 | 1 |
| 22 (Knäuper et al., 2004) | (Knäuper et al., 2004c) | | PUB | STU | 111 | 48.6 | Eating-related risk behavior | CS | Specific | Diet | 0.66 | 1 | 0 | 0 |
| 22 (Knäuper et al., 2004) | (Knäuper et al., 2004d) | | PUB | STU | 111 | 48.6 | Weight regulation risk behavior | CS | Specific | Diet | 0.57 | 1 | 0 | 0 |
| 26 (Lavins, 2013) | (Lavins, 2013) | | UPUB | STU | 212 | 51.9 | Health Behavior Checklist | CS | General | — | 0.752 | 0 | 1 | 0 |
| 28 (Merillat & Gonzalez-Vallejo, 2019) | (Merillat & Gonzalez-Vallejo, 2019) | | PUB | GP | 217 | 69.6 | Health Behavior Checklist | CS | General | — | 0.8 | 0 | 1 | 0 |
| 29 (Miquelon et al., 2012) | (Miquelon et al., 2012) | | PUB | CLIN | 119 | 100 | Weight-loss dieting | LT | Specific | Diet | 0.68 | 0 | 0 | 0 |
| 32 (Natrop, 2015) | (Natrop, 2015a) | | UPUB | STU | 139 | 64 | Healthier eating or more exercise | CS | Specific | — | 0.85 | 0 | 0 | 0 |
| 32 (Natrop, 2015) | (Natrop, 2015b) | | UPUB | STU | 139 | 64 | Fruit and vegetable consumption | CS | Specific | Diet | 0.85 | 0 | 0 | 0 |
| 33 (Neufeld, 2015) | (Neufeld, 2015a) | | UPUB | STU | 139 | 64 | Physical activity and diet | CS | Specific | PA | 0.66 | 0 | 0 | 0 |
| 33 (Neufeld, 2015) | (Neufeld, 2015b) | | UPUB | STU | 139 | 64 | Physical activity | CS | Specific | PA | 0.66 | 0 | 0 | 0 |
| 33 (Neufeld, 2015) | (Neufeld, 2015c) | | UPUB | STU | 139 | 64 | Physical fitness | CS | Specific | PA | 0.66 | 0 | 0 | 0 |
| 37 (Pink et al., 2022) | (Pink et al., 2022) | | PUB | STU | 148 | 54.7 | Physical activity | CS | General | PA | 0.8 | 0 | 0 | 0 |
| 38 (Prinsen, 2017) | (Prinsen, 2017a) | | UPUB | STU | 330 | 72.7 | Physical activity | CS | General | PA | 0.88 | 0 | 0 | 0 |
| 38 (Prinsen, 2017) | (Prinsen, 2017b) | | UPUB | STU | 330 | 72.7 | Physical activity | CS | Specific | PA | 0.78 | 0 | 0 | 0 |
| 41 (Radtke & Kaklamanou et al., 2014) | (Radtke & Kaklamanou et al., 2014) | | PUB | STU | 75 | 100 | Diet | CS | Specific | Diet | 0.44 | 1 | 1 | 1 |
| 42 (Radtke & Rackow, 2014) | (Radtke & Rackow, 2014) | | PUB | STU | 135 | 51.5 | Physical activity (Taking the stairs) | CS | Specific | PA | 0.84 | 0 | 0 | 0 |
| 43 (Radtke & Scholz, 2017) | (Radtke & Scholz, 2017b) | | PUB | STU | 227 | 61.7 | Healthy lifestyle | CS | Specific | — | 0.93 | 0 | 0 | 0 |
| 47 (Scheffels, 2016) | (Scheffels, 2016a) | | UPUB | STU | 209 | 63 | Physical activity | CS | Specific | PA | 0.73 | 0 | 0 | 0 |
| 47 (Scheffels, 2016) | (Scheffels, 2016b) | | UPUB | STU | 209 | 63 | Physical activity | CS | General | PA | 0.79 | 0 | 0 | 0 |
| 48 (Selten, 2012) | (Selten, 2012) | | UPUB | GP | 597 | 100 | Exercise | CS | Specific | — | 0.58 | 1 | 0 | 0 |
| 50 (Smoletz, 2016) | (Smoletz, 2016a) | | UPUB | STU | 195 | 58.5 | Physical activity | CS | General | PA | 0.7 | 0 | 0 | 0 |
| 50 (Smoletz, 2016) | (Smoletz, 2016b) | | UPUB | STU | 195 | 58.5 | Physical activity | CS | Specific | PA | 0.81 | 0 | 0 | 0 |
| 51 (Spranger, 2014) | (Spranger, 2014a) | | UPUB | STU | 123 | 65.3 | Diet | LT | Specific | Diet | 0.78 | 0 | 0 | 0 |
| 51 (Spranger, 2014) | (Spranger, 2014b) | | UPUB | STU | 112 | 65.3 | Diet | LT | Specific | Diet | 0.78 | 0 | 0 | 0 |
| 51 (Spranger, 2014) | (Spranger, 2014c) | | UPUB | STU | 100 | 65.3 | Diet | LT | Specific | Diet | 0.81 | 0 | 0 | 0 |
| 56 (Thongworn & Sirisuk, 2018) | (Thongworn & Sirisuk, 2018) | | PUB | STU | 788 | 77.7 | Implementation of weight-control behavior | CS | Specific | PA | 0.88 | 1 | 0 | 0 |
| 57 (West et al., 2017) | (West et al., 2017) | | PUB | STU | 119 | 47.1 | Physical activity | CS | Specific | PA | 0.78 | 0 | 0 | 0 |
| 58 (Zhou et al., 2016) | (Zhou et al., 2016c) | | PUB | GP | 140 | 40 | Slow down while making/answering calls | CS | General | — | 0.91 | 0 | 0 | 0 |
| 58 (Zhou et al., 2016) | (Zhou et al., 2016d) | | PUB | GP | 140 | 40 | Pull over to the side of the road when making/answering calls | CS | General | — | 0.91 | 0 | 0 | 0 |
| 58 (Zhou et al., 2016) | (Zhou et al., 2016e) | | PUB | GP | 140 | 40 | Increase following distance when making/answering calls | CS | General | — | 0.91 | 0 | 0 | 0 |
| 58 (Zhou et al., 2016) | (Zhou et al., 2016f) | | PUB | GP | 140 | 40 | Change lanes less frequently while making/answering calls | CS | General | — | 0.91 | 0 | 0 | 0 |
| 58 (Zhou et al., 2016) | (Zhou et al., 2016g) | | PUB | GP | 140 | 40 | Shorten conversations while making/answering calls | CS | General | — | 0.91 | 0 | 0 | 0 |
| 58 (Zhou et al., 2016) | (Zhou et al., 2016h) | | PUB | GP | 140 | 40 | Remind the caller that he/she is driving | CS | General | — | 0.91 | 0 | 0 | 0 |
| 58 (Zhou et al., 2016) | (Zhou et al., 2016i) | | PUB | GP | 140 | 40 | Refuse to answer the call | CS | General | — | 0.91 | 0 | 0 | 0 |
| 58 (Zhou et al., 2016) | (Zhou et al., 2016l) | | PUB | GP | 140 | 40 | Slow down when reading a message | CS | General | — | 0.91 | 0 | 0 | 0 |
| 58 (Zhou et al., 2016) | (Zhou et al., 2016m) | | PUB | GP | 140 | 40 | Pull over to the side of the road when reading a message | CS | General | — | 0.91 | 0 | 0 | 0 |
| 58 (Zhou et al., 2016) | (Zhou et al., 2016n) | | PUB | GP | 140 | 40 | Increase following distance when reading a message | CS | General | — | 0.91 | 0 | 0 | 0 |
| 58 (Zhou et al., 2016) | (Zhou et al., 2016o) | | PUB | GP | 140 | 40 | Change lanes less frequently when reading a message | CS | General | — | 0.91 | 0 | 0 | 0 |
| 58 (Zhou et al., 2016) | (Zhou et al., 2016pc) | | PUB | GP | 140 | 40 | Slow down while making/answering calls | CS | Specific | — | 0.58 | 0 | 0 | 0 |
| 58 (Zhou et al., 2016) | (Zhou et al., 2016pd) | | PUB | GP | 140 | 40 | Pull over to the side of the road when making/answering calls | CS | Specific | — | 0.58 | 0 | 0 | 0 |
| 58 (Zhou et al., 2016) | (Zhou et al., 2016pe) | | PUB | GP | 140 | 40 | Increase following distance when making/answering calls | CS | Specific | — | 0.58 | 0 | 0 | 0 |
| 58 (Zhou et al., 2016) | (Zhou et al., 2016pf) | | PUB | GP | 140 | 40 | Change lanes less frequently while making/answering calls | CS | Specific | — | 0.58 | 0 | 0 | 0 |
| 58 (Zhou et al., 2016) | (Zhou et al., 2016pg) | | PUB | GP | 140 | 40 | Shorten conversations while making/answering calls | CS | Specific | — | 0.58 | 0 | 0 | 0 |
| 58 (Zhou et al., 2016) | (Zhou et al., 2016ph) | | PUB | GP | 140 | 40 | Remind the caller that he/she is driving | CS | Specific | — | 0.58 | 0 | 0 | 0 |
| 58 (Zhou et al., 2016) | (Zhou et al., 2016pi) | | PUB | GP | 140 | 40 | Refuse to answer the call | CS | Specific | — | 0.58 | 0 | 0 | 0 |
| 58 (Zhou et al., 2016) | (Zhou et al., 2016pl) | | PUB | GP | 140 | 40 | Slow down when reading a message | CS | Specific | — | 0.58 | 0 | 0 | 0 |
| 58 (Zhou et al., 2016) | (Zhou et al., 2016pm) | | PUB | GP | 140 | 40 | Pull over to the side of the road when reading a message | CS | Specific | — | 0.58 | 0 | 0 | 0 |
| 58 (Zhou et al., 2016) | (Zhou et al., 2016pn) | | PUB | GP | 140 | 40 | Increase following distance when reading a message | CS | Specific | — | 0.58 | 0 | 0 | 0 |
| 58 (Zhou et al., 2016) | (Zhou et al., 2016po) | | PUB | GP | 140 | 40 | Change lanes less frequently when reading a message | CS | Specific | — | 0.58 | 0 | 0 | 0 |

*Note.* Study ID = Identifier for independent samples; Effect Size ID = Identifier of a specific correlation between a given CHB and a single behavioral outcome; CHB = Compensatory health belief; PUB = Published; UPUB = Unpublished; STU = Student; GP = General population; CLIN = Clinical; CS = Cross-sectional; LT = Longitudinal; Alpha = Cronbach’s α. For continuous moderator coding, 0 = Absent; 1 = Present

*Indicates that the student sample was 8^th^-9^th^ grade students rather than college students which make up the rest of the student sample.

**Supplementary Figure 1.**

*Hypothesized relationships among CHBs with Intentions and Behaviors*

**
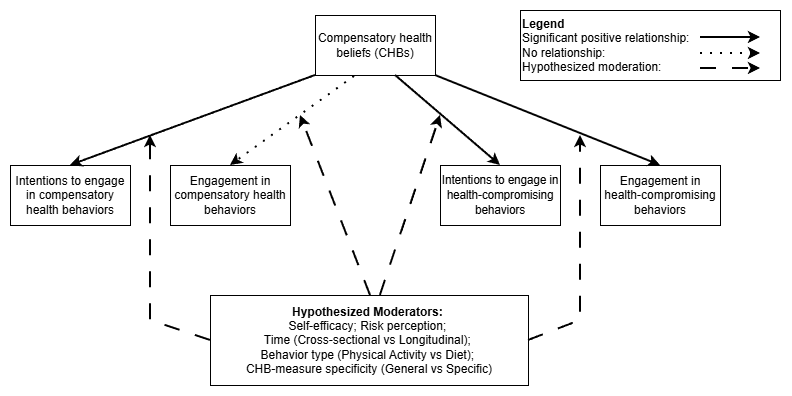
**

**Supplementary Figure 2.**

*Funnel Plots of Primary Analyses*


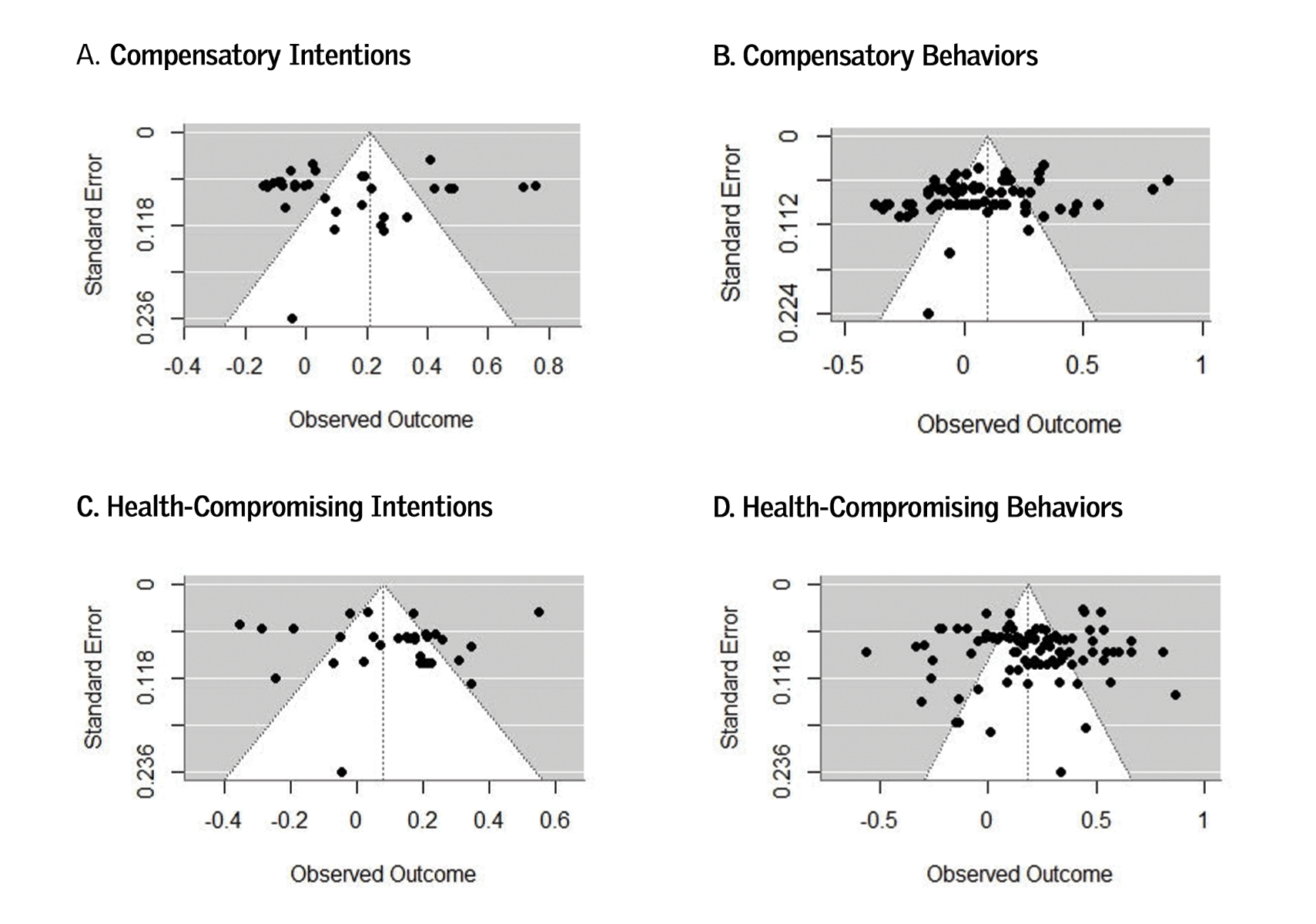


*Note.* Each funnel plot is centered around the mean effect size for the relationship between CHBs and the corresponding behavioral outcome.
