# Supplementary material for: Vices and vegetables: a systematic review and series of meta-analyses examining the relationships among compensatory health beliefs with health-related intentions and behaviors
Source: Ann Behav Med. 2026 Apr 19;60(1):kaag013. doi: 10.1093/abm/kaag013 (PMC13092134; doi:10.1093/abm/kaag013)
Supplement: kaag013_Supplementary_Data [file kaag013_supplementary_data.zip › References for Studies Included - 3.13.26.docx]

# **References for Studies Included in Meta-analyses**

1. Amrein, M. A., Rackow, P., Inauen, J., Radtke, T., & Scholz, U. (2017). The role of compensatory health beliefs in eating behavior change: A mixed method study. *Appetite*, *116*, 1–10. https://doi.org/10.1016/j.appet.2017.04.016
2. An, Y., & Zhang, M. X. (2024). Relationship between problematic smartphone use and sleep problems: The roles of sleep-related compensatory health beliefs and bedtime procrastination. *Digital Health*, *10*. https://doi.org/10.1177/20552076241283338
3. Austermann, M. (2016). *Explanations for substance use- the role of implicit and explicit compensatory health beliefs and personality for alcohol/nicotine-related risk behavior*.
4. Berli, C., Loretini, P., Radtke, T., Hornung, R., & Scholz, U. (2014). Predicting physical activity in adolescents: The role of compensatory health beliefs within the Health Action Process Approach. *Psychology and Health*, *29*(4), 458–474. https://doi.org/10.1080/08870446.2013.865028
5. Duan, Y., Liang, W., Wang, Y., Hu, C., & Lippke, S. (2024). Modelling the compensatory and carry-over effects between physical activity and fruit-vegetable consumption in young adults. *Scientific Reports*, *14*(1). https://doi.org/10.1038/s41598-024-81585-7
6. Ernsting, A., Schwarzer, R., Lippke, S., & Schneider, M. (2013). “I do not need a flu shot because I lead a healthy lifestyle”: Compensatory health beliefs make vaccination less likely. *Journal of Health Psychology*, *18*(6), 825–836. https://doi.org/10.1177/1359105312455076
7. Finlay, A. H., Boyland, E. J., Jones, A., Langfield, T., Bending, E., Malhi, M. S., & Robinson, E. (2024). Passive overconsumption? Limited evidence of compensation in meal size when consuming foods high in energy density: Two randomised crossover experiments. *Appetite*, *200*, 1–12. https://doi.org/10.1016/j.appet.2024.107533
8. Fleig, L., Ngo, J., Roman, B., Ntzani, E., Satta, P., Warner, L. M., Schwarzer, R., & Brandi, M. L. (2015). Beyond single behaviour theory: Adding cross-behaviour cognitions to the health action process approach. *British Journal of Health Psychology*, *20*(4), 824–841. https://doi.org/10.1111/bjhp.12144
9. Forestier, C., Sarrazin, P., Sniehotta, F., Allenet, B., Heuzé, J. P., Gauchet, A., & Chalabaev, A. (2020). Do compensatory health beliefs predict behavioural intention in a multiple health behaviour change context? Evidence in individuals with cardiovascular diseases? *Psychology, Health and Medicine*, *25*(5), 593–600. https://doi.org/10.1080/13548506.2019.1653476
10. Fox, G.Q. (Unpublished Data) *How does changing one health behavior impact another health behavior? Investigating compensation effects.*
11. Gallagher, K. M. (2019). What do we know about the health of first-generation college students? A first look at compensatory health beliefs and behavior. *Perspectives In Learning*, *18*(1), 37–45. https://csuepress.columbusstate.edu/pil
12. Glock, S., Müller, B. C. N., & Krolak-Schwerdt, S. (2013). Implicit associations and compensatory health beliefs in smokers: Exploring their role for behaviour and their change through warning labels. *British Journal of Health Psychology*, *18*(4), 814–826. https://doi.org/10.1111/bjhp.12023
13. Gough, T., Brealey, J., Finlay, A., Jones, A., & Robinson, E. (2025). The effect of stealth vs. declared reductions to lunch meal portion size on subsequent energy intake: A randomised control experiment. *Food Quality and Preference*, *127*, 1–8. https://doi.org/10.1016/j.foodqual.2025.105443
14. Hartmann, C., Keller, C., & Siegrist, M. (2016). Compensatory beliefs, nutrition knowledge and eating styles of users and non-users of meal replacement products. *Appetite*, *105*, 775–781. https://doi.org/10.1016/j.appet.2016.07.013
15. Hein, S. (2014). *Compensatory health beliefs and behaviors on alcohol consumption versus the theory of planned behavior*.
16. Heinz, S. (2013). *The role of compensatory health beliefs and personality structure in exercise behavior*.
17. Hoffman, R. K. (2023). Testing cognitive dissonance as a mechanism behind compensatory behaviors in the context of heavy college drinking. In *Santa Barbara M.A. in Applied Social Psychology*.
18. Kaklamanou, D., & Armitage, C. J. (2012). Testing compensatory health beliefs in a UK population. *Psychology and Health*, *27*(9), 1062–1074. https://doi.org/10.1080/08870446.2012.662974
19. Klein, A.-C. (2017). *Exploring the new constructs of exercising: How exercise related obsessive-compulsiveness, compensatory health beliefs (CHB’s) and outcome expectations are related to exercise addiction and normal exercise*.
20. Knäuper, B., Rabiau, M., Cohen, O., & Patriciu, N. (2004). Compensatory health beliefs: Scale development and psychometric properties. *Psychology and Health*, *19*(5), 607–624. https://doi.org/10.1080/0887044042000196737
21. Kronick, I., Auerbach, R. P., Stich, C., & Knäuper, B. (2011). Compensatory beliefs and intentions contribute to the prediction of caloric intake in dieters. *Appetite*, *57*(2), 435–438. https://doi.org/10.1016/j.appet.2011.05.306
22. Kronick, I., & Knäuper, B. (2010). Temptations elicit compensatory intentions. *Appetite*, *54*(2), 398–401. https://doi.org/10.1016/j.appet.2009.12.011
23. Kruse, D. (2015). *The creation of compensatory health beliefs through positive and negative alcohol specific commercials : A survey experiment*.
24. Lavins, B. D. (2013). *Testing the compensatory health belief scale: The role of cognitive factors and their relationship to health outcomes*.
25. Matley, F. A. I., & Davies, E. L. (2018). Resisting temptation: Alcohol specific self-efficacy mediates the impacts of compensatory health beliefs and behaviours on alcohol consumption. *Psychology, Health and Medicine*, *23*(3), 259–269. https://doi.org/10.1080/13548506.2017.1363395
26. Merillat, B. D., & González-Vallejo, C. (2019). Compensatory health beliefs relate to decision-making coherence and health patterns. *Ohio Journal of Science*, *119*(2), 79–91. https://doi.org/10.18061/ojs.v119i2.6709
27. Miquelon, P., Knäuper, B., & Vallerand, R. J. (2012). Motivation and goal attainment. The role of compensatory beliefs. *Appetite*, *58*(2), 608–615. https://doi.org/10.1016/j.appet.2011.12.025
28. Moll, A. (2014). *Does the holding of compensatory health beliefs correlate with willingness to engage in indoor / salon tanning amongst 18-25 year olds?*
29. Nasser, J. D. (2016). *Dietary restraint in individuals with symptoms of binge eating disorder: Manifestation and its relation to binge eating behavior*.
30. Natrop, L. (2015). *Reasons for unhealthy eating - How compensatory health beliefs and personality are related to eating behavior*.
31. Neufeld, M. (2015). *The relation between personality and compensatory health beliefs and-behavior concerning regular physical activity*.
32. Oberschmidt, K. (2017). *The relationship between binge-watching, compensatory health beliefs, and sleep*.
33. Olding, T. (2018). *Psychological consequences and antecedents of binge-watching in young adults*. 1–27.
34. Oviedo Ramirez, S. L. (2017). *The development of a drinking-specific compensatory health beliefs scale*. https://digitalcommons.utep.edu/open_etd/518
35. Paulus, A. M., & Aziz, A. (2023). Binge watching, compensatory health beliefs and academic procrastination among university students. *Journal of Behavioral Sciences*, *33*(1), 2023.
36. Pink, A. E., Lim, P. X. H., Sim, A. Y., & Cheon, B. K. (2022). The effects of acute social media exposure on body dissatisfaction and eating behavior of male and female students. *Journal of Social and Clinical Psychology*, *41*(4), 365–397. https://doi.org/10.1521/jscp.2022.41.4.365
37. Prinsen, M. (2017). *The association of compensatory health beliefs with binge-watching, physical activity and body mass index in young adults*.
38. Rabiau, M. A., Knäuper, B., Nguyen, T. K., Sufrategui, M., & Polychronakos, C. (2009). Compensatory beliefs about glucose testing are associated with low adherence to treatment and poor metabolic control in adolescents with type 1 diabetes. *Health Education Research*, *24*(5), 890–896. https://doi.org/10.1093/her/cyp032
39. Radtke, T., Inauen, J., Rennie, L., Orbell, S., & Scholz, U. (2014). Trait versus state effects of dispositional and situational compensatory health beliefs on high-calorie snack consumption. *Zeitschrift Fur Gesundheitspsychologie*, *22*(4), 156–164. www.hogrefe.de/zeitschriften/zgp4/14
40. Radtke, T., Kaklamanou, D., Scholz, U., Hornung, R., & Armitage, C. J. (2014). Are diet-specific compensatory health beliefs predictive of dieting intentions and behaviour? *Appetite*, *76*, 36–43. https://doi.org/10.1016/j.appet.2014.01.014
41. Radtke, T., & Rackow, P. (2014). Autonomous motivation is not enough: The role of compensatory health beliefs for the readiness to change stair and elevator use. *International Journal of Environmental Research and Public Health*, *11*(12), 12412–12428. https://doi.org/10.3390/ijerph111212412
42. Radtke, T., & Scholz, U. (2017). Beliefs are not behavior: The distinction between compensatory health beliefs and compensatory health behavior. *Zeitschrift Fur Gesundheitspsychologie*, *24*(3), 119–129. https://doi.org/10.1026/0943-8149/a000161
43. Radtke, T., Scholz, U., Keller, R., & Hornung, R. (2012). Smoking is ok as long as I eat healthily: Compensatory health beliefs and their role for intentions and smoking within the Health Action Process Approach. *Psychology and Health*, *27*(SUPPL. 2), 91–107. https://doi.org/10.1080/08870446.2011.603422
44. Ricker, C. (2013). *The development of an alcohol-specific compensatory health belief scale*.
45. Scheffels, J. (2016). *The role of physical activity in the relation between compensatory health beliefs and alcohol consumption among young adults*.
46. Selten, E. (2012). *The formation of compensatory intentions after overeating*.
47. Sim, A. Y., & Cheon, B. K. (2019). Influence of impending healthy food consumption on snacking: Nudging vs. compensatory behaviour. *Physiology and Behavior*, *198*, 48–56. https://doi.org/10.1016/j.physbeh.2018.10.010
48. Smoletz, F. (2016). *Unhealthy eating: How sport and stress are related to eating-specific compensatory health behavior and beliefs*.
49. Spranger, I. (2014). *An exploratory study on the relation between compensatory health beliefs and behavior*.
50. Storm, V., Reinwand, D., Wienert, J., Kuhlmann, T., de Vries, H., & Lippke, S. (2017). Brief report: Compensatory health beliefs are negatively associated with intentions for regular fruit and vegetable consumption when self-efficacy is low. *Journal of Health Psychology*, *22*(8), 1094–1100. https://doi.org/10.1177/1359105315625358
51. Tăut, D., & Băban, A. (2008). Examination of the relationship between self-regulatory strategies and healthy eating patterns in coronary heart disease patients. The relevance of compensatory health beliefs. *Cognition,Brain,Behavior*, *12*(2), 219–231.
52. te Wilde, E. (2013). *Compensatory health beliefs-the development of an eating-specific scale*.
53. Thomas, L. (2016). *The impact of personality on compensatory health beliefs: Does the theory of planned behavior play a mediating role?*
54. Thongworn, S., & Sirisuk, V. (2018). Weight control specific compensatory health beliefs: Hypothetical testing and model extension. *Kasetsart Journal of Social Sciences*, *39*(2), 312–319. https://doi.org/10.1016/j.kjss.2017.06.006
55. West, J., Guelfi, K. J., Dimmock, J. A., & Jackson, B. (2017). “I deserve a treat”: Exercise motivation as a predictor of post-exercise dietary licensing beliefs and implicit associations toward unhealthy snacks. *Psychology of Sport and Exercise*, *32*, 93–101. https://doi.org/10.1016/j.psychsport.2017.06.007
56. Zhou, R., Yu, M., & Wang, X. (2016). Why do drivers use mobile phones while driving? The contribution of compensatory beliefs. *PLoS ONE*, *11*(8). https://doi.org/10.1371/journal.pone.0160288
57. Zhou, R., Zhang, Y., & Shi, Y. (2020). Driver’s distracted behavior: The contribution of compensatory beliefs increases with higher perceived risk. *International Journal of Industrial Ergonomics*, *80*, 1–12. https://doi.org/10.1016/j.ergon.2020.103009
